# 25th International Symposium on Infections in the Critically Ill Patient

**DOI:** 10.3390/medsci8010013

**Published:** 2020-02-13

**Authors:** Antonio Artigas, Jean Carlet, Ricard Ferrer, Michael Niederman, Antoni Torres

**Affiliations:** 1 Critical Care Center, Sabadell Hospital, University Institute Parc Taulí, Autonomous University of Barcelona, 08193 Ciberes, Spain; 2 President of the World Alliance against Antibiotic Resistance (WAAAR), 75008 Paris, France; 3 Intestive Care Medicine Department, Vall d’Hebron University Hospital, 08035 Barcelona, Spain; 4 Division of Pulmonary and Critical Care Medicine, New York Presbyterian Hospital, Weill Cornell Medical College, New York, NY 10065, USA; 5 Pulmonology Department, Clinic Hospital, University of Barcelona, CIBER Enfermedades Respiratorias, 08036 Barcelona, Spain


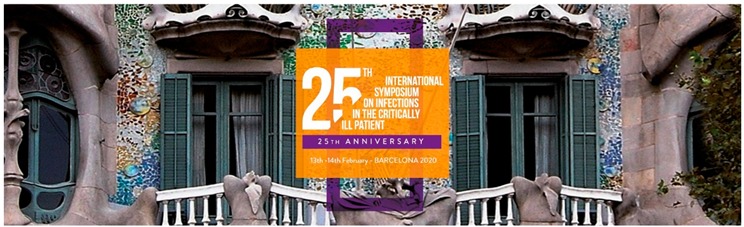


## 1. Introduction

This 25th International Symposium on Infections in the Critically Ill Patient aims to review current concepts, technology and present advances in infections in critically ill patient. Sepsis, Pulmonary Infections and their therapeutic and preventive strategies will be the topics presented by international experts who will review and update sepsis as a global international problem.

New guidelines epidemiological information on sepsis, fluid therapy and vasopressors, a personalize sepsis care and new therapies and future randomized control trials are provided. The immune response and the emerging methods to personalize sepsis care including new biomarkers, sepsis phenotypes and end types and immunomonitoring of patients with sepsis represent a new complementary view to treat patients with severe infections and organ failure in addition to early antibiotic and the control of source of infection. New insights of cell therapies and extracorporeal treatment will be provided.

The preliminary information about the European severe community pneumonia guidelines and new diagnostic approaches for nosocomial pulmonary infections, new antibiotics and the optimization of their use represent key factors to improve outcome and prevention of severe infections in the critically ill patients.
**Antonio Artigas, MD** Corporación Sanitaria Universitaria Parc Tauli CIBER Enfermedades Respiratorias Autonomous University of Barcelona Barcelona, Spain**Ricard Ferrer, MD** Intensive Care Department University Hospital Vall d'Hebron CIBER Enfermedades Respiratorias Barcelona, Spain**Antonio Torres, MD** Pneumology Department Clinic Hospital University of Barcelona CIBER Enfermedades Respiratorias Barcelona, Spain

## 2. Abstracts Speakers

### 2.1. SESION I. SEPSIS

#### **2.1.1. How 25 Years of Research Has Changed My Practice in Sepsis?** 

VincentJean-LouisDept of Intensive care, Erasme Hospital, Université libre de Bruxelles, Brussels, Belgium

Despite decades of sepsis research, no specific immunomodulatory sepsis therapies are currently available, except perhaps corticosteroids, so from that point of view, research has not changed my clinical practice very much. However, research and changes in other aspects of patient management and process of care have certainly influenced the way I treat my patients with sepsis. There are perhaps three key areas that have altered over the years:

1. One of the most important realizations has been the importance of the time factor when managing patients with sepsis −time is tissue. Making a rapid diagnosis and starting appropriate treatment promptly are keys to limiting organ dysfunction and maximizing patient outcomes. Increased awareness of sepsis in the ICU but also in other hospital departments and before hospital admission is helping identify patients earlier so that appropriate monitoring, investigations and treatment can be started. Rapid response teams that can attend patients with suspected sepsis on the general ward are now present in many hospitals. The need for adequate, rapid resuscitation has also been highlighted and increasingly fluids and vasopressors are started simultaneously rather than waiting to see what effect may fluid has before vasoactive agents are introduced. Any period of hypotension can be harmful, and it is better to start these two branches of resuscitation together to restore a minimum perfusion pressure as soon as possible. Vasopressor agents can then be weaned once the patient’s hemodynamic status is optimized. An important research result in the last 25 years was the finding that norepinephrine should be used in preference to dopamine as the first-line vasopressor. The importance of removal of excess fluid once the patient has stabilized has also come to the fore with persistent positive fluid balance associated with worse outcomes. My management of the patient with sepsis is thus guided by the SOSD paradigm: salvage, optimization, stabilization, de-escalation. I use changes in blood lactate levels over time to provide an indication of response to treatment, with increasing or stable levels suggesting diagnosis and/or treatment needs to be reviewed and possibly altered. 

2. Another area where practice has changed is that we have become much less invasive. Echography is much more widely available and practiced routinely at the bedside in many units for initial hemodynamic assessment and diagnosis of associated cardiac conditions. The pulmonary artery catheter still has a place in severely ill, complex patients but non-invasive techniques have replaced it in some patients. The move towards less-invasive monitoring is set to continue as technology advances and devices become smaller, more effective and efficient, and more available. 

3. A third area, the need to personalize therapies, has also been stressed in recent years and is reflected in using fewer one-size-fits-all targets and adapting treatments according to the characteristics and needs of individual patients. As just one example, a target mean arterial pressure of 65 mmHg may be appropriate for many patients, but a patient with chronic hypertension may benefit from a higher target, and in a younger patient with no evidence of arteriosclerosis, a lower pressure may be adequate. Similarly, blood transfusions should not be given based only on a hemoglobin concentration but the pros and cons weighed up in each patient, taken into consideration age, disease severity, and a history of cardiac ischemia among others. 

As we become more able to characterize the sepsis response in individual patients, we will be better able to select patients for inclusion in clinical trials according to their likelihood to respond to treatment rather than testing all agents in heterogeneous groups as was the rule until recently. This will result in more trial interventions demonstrating a beneficial effect on survival and I am confident that within the next 25 years, I will be including personalized immunomodulatory therapies in the management of my patients with sepsis. 

Suggested reading

- Vincent JL (2018) How I treat septic shock. Intensive Care Med 44:2242–2244.
- Annane D, Renault A, Brun-Buisson C, et al. (2018) Hydrocortisone plus fludrocortisone for adults with septic shock. N Engl J Med 378:809–818
- Rhodes A, Evans LE, Alhazzani W et al. (2017) Surviving Sepsis Campaign: International Guidelines for Management of Sepsis and Septic Shock: 2016. Crit Care Med 45:486–552.
- De Backer D, Aldecoa C, Njimi H et al. (2012) Dopamine versus norepinephrine in the treatment of septic shock: A metaanalysis. Crit Care Med 40:725–730.
- Saugel B, Vincent JL, Wagner JY (2017) Personalized hemodynamic management. Curr Opin Crit Care 23:334–341

#### **2.1.2. Are Sepsis Outcomes Predetermined** 

FerrerRicardIntensive Care Medicine Department, Vall d’Hebron University Hospital, Barcelona and SpainAlong with the control of source of infection and adequate hemodynamic resuscitation, the administration of an early appropriate antimicrobial treatment is the single the most important available means to face the adverse prognosis associated with sepsis. Sepsis is a medical emergency and time-dependent condition due to a dysregulated host response to infection, which is associated with multi-organ dysfunction and unacceptably high odds for mortality. Based on this premise, when treating patients with possible or confirmed sepsis, clinicians must initiate broad-spectrum antimicrobials as soon as possible, preferably within the first hour of diagnosis, as recommended by the 2016 SSC guidelines. Different scientific societies give support for the importance of timing, as several studies have demonstrated the benefits of the early initiation of appropriate antibiotic therapy, particularly in patients with septic shock. Accordingly, there are many well-performed studies on the effects of delayed administration of antibiotics in patients with sepsis and septic shock. Delayed administration of antibiotics has been also associated with increased hospital length of stay, higher rates of acute lung injury and acute kidney injury, and worsening organ dysfunction. However, the effective application of institutional protocols to improve the administering antimicrobials within 1 h from presentation remains challenging. Availability of antimicrobials, general disbeliefs regarding the severity of sepsis and inappropriate local policies are some barriers to implement suitable goals. As medical decisions rely on clinical judgment, when facing a patient presenting with a non-severe infection or an alternative diagnosis is being considered, additional diagnostic data should be obtained before administering broad-spectrum antibiotics. Optimizing antimicrobial therapy is essential to reduce mortality and the emergence of multi-drug resistant pathogens. The routine assessment of the previous risk for multi-drug resistant pathogens prior to the early administration of antimicrobials should be performed in all patients. Procalcitonin-based approaches have been demonstrated to be useful to guide treatment in most patients with sepsis and septic shock. Compliance to practice guidelines, optimization and continuous assessment of duration and appropriateness of antimicrobials are essential components of any antimicrobial stewardship program. De-escalation and shortened courses of antimicrobials are also key components of such programs.

##### **References** 

1. Rhodes, A.; Evans, L.E.; Alhazzani, W.; Levy, M.M.; Antonelli, M.; Ferrer, R.; Kumar, A.; Sevransky, J.E.; Sprung, C.L.; Nunnally, M.E.; et al. Surviving Sepsis Campaign: International Guidelines for Management of Sepsis and Septic Shock: 2016. *Intensiv. Care Med.*
**2017**, *43*, 304–377.
2. Kumar, A.; Roberts, D.; Wood, K.E.; Light, B.; Parrillo, J.E.; Sharma, S.; Suppes, R.; Feinstein, D.; Zanotti, S.; Taiberg, L.; et al. Duration of hypotension before initiation of effective antimicrobial therapy is the critical determinant of survival in human septic shock*. *Crit. Care Med.*
**2006**, *34*, 1589–1596.
3. Seymour, C.W.; Gesten, F.; Prescott, H.C.; Friedrich, M.E.; Iwashyna, T.J.; Phillips, G.S.; Lemeshow, S.; Osborn, T.; Terry, K.M.; Levy, M.M. Time to Treatment and Mortality during Mandated Emergency Care for Sepsis. *N. Engl. J. Med.*
**2017**, *376*, 2235–2244.
4. Bloos, F.; Rüddel, H.; Thomas-Rüddel, D.; Schwarzkopf, D.; Pausch, C.; Harbarth, S.; Schreiber, T.; Gründling, M.; Marshall, J.; Simon, P.; et al. Effect of a multifaceted educational intervention for anti-infectious measures on sepsis mortality: A cluster randomized trial. *Intensive Care Med*. **2017**, *43*, 1602–1612.
5. Plata-Menchaca, E.; Esteban, E.; Ferrer, R. Antimicrobial Stewardship in Sepsis. In *Annual Update in Intensive Care and Emergency Medicine*; Springer Science and Business Media LLC, 2019; pp. 507–519.

#### **2.1.3. We Must Act Before the Hospital Admission** 

GerlachHerwigDept. for Anesthesia, Intensive Care, Medicine and Pain Management Vivantes—Klinikum Neukoelln, Rudower Strasse 48, D-12351 Berlin, Germany

“Hectic fever at its inception is difficult to recognize but easy to treat; left untended, it becomes easy to recognize but difficult to treat.” This historical citation from Niccolò Machiavelli around 1513 AC [1] seems very simple but contains a lot of hints leading to issues which are part of current discussions about (very) early treatment of sepsis and septic shock. Looking back, the structured research on the ideal timepoint of sepsis treatment began in 2004, when the Surviving Sepsis Campaign (SSC) published the first international guidelines on the management of sepsis. Meanwhile, the 4th version of the SSC guidelines are available, and several recommendations contain a defined timepoint [2].

Based on the initial guidelines, the SSC launched a prospective project testing so called “Sepsis Bundles”, which are a condensed form of some (not all) recommendations from the guidelines. One of these bundles (called “Sepsis Resuscitation Bundle”) has a time frame of 6 h after identifying severe sepsis or septic shock; the second (called “Sepsis Management Bundle”) was limited to 24 h. In 2010, the data from this project were published [3]; it was demonstrated that several treatment options had a clear time-dependent effect, one of which was the early application of antibiotics. In contrast, other treatments failed to show a benefit. Hence, the SSC bundles were reviewed, resulting in a single bundle containing early treatments with a time frame of 3 or 6 h. Based on a political action, this bundle was initiated as a mandatory rule for hospitals in several states of the USA. 

In 2017, the electronic hospitals records for these states were evaluated regarding the effect of the sepsis bundle on the outcome of included patients [4]. The data revealed a time-dependent survival benefit of early administration of antibiotics, early measurement of lactate, as well as early blood cultures in septic patients. All three options demonstrated their positive effect continuously over time and from the first hour after sepsis detection. This led the SSC to revise the bundles again, which resulted in the recent SSC Bundles 2018 [5]; based on the aforementioned results, the time frame was now set to 60 min for the treatment options 1) blood cultures, 2) lactate measurement, 3) application of broad-spectrum antibiotics, 4) fluid bolus in patients with hypotension or lactate > 4 mmol/L, and 5) administration of vasopressors after the fluid bolus if hypotension is still present to maintain a mean arterial pressure of ≥65 mmHg [5]. Briefly after the aforementioned 1-h bundle was published, there was a huge discussion, partially by internet-based petitions, and finally it was decided to put the new 1-h bundle “on hold”. 

The discussions, which are still ongoing, are partially very puzzling. One major issue is the early administration of broad-spectrum antibiotics, which is questioned especially by infectiologists, who express their worry about unneeded treatment in patients without the final diagnosis of sepsis. Moreover, they claim that the 1-h bundle may lead to more antibiotics use in total, which is definitely against the current trend to stop the overuse of antibiotics in hospitals. A recent Pro-Con-debate tried to find a compromise but finally failed to do so [6,7]: The Authors “Pro Early Antibiotics” cited several studies including the aforementioned paper by Seymour et al. [4], which together provide a very convincing picture on the obvious benefit of early antibiotics even within one hour in patients with sepsis or septic shock. Unfortunately, the Authors “Con Early Antibiotics” did not challenge these facts but concentrated completely on the terms “appropriate” and “broad-spectrum” for antibiotics. One argument in this context is, that these two descriptors are not synonymous, and that appropriate empiric therapy should consist of targeted antibiosis that considers the unique factors and context of the patient [7]. This leads back to the initial citation of Machiavelli from the 16th century: without clear recognition of “unique factors” and “context of the patient”, which is a synonym for an *unambiguous diagnosis*, no treatment or treatment that is too late?! But this is exactly the wrong way! We have to accept that we are simply not able to make a clear diagnosis in the very early phase of sepsis management, and that this sensitive phase is the optimal time point for effective antibiosis.

Finally, what is with the prehospital setting? Data are very limited, and there are some ongoing studies to evaluate the effect of treatment bundles in this phase. Most of these studies concentrate on the recognition of patients, which is the key issue in the very early phase of sepsis, as we discussed already above. In a small study in mobile emergency units, a point-of-care lactate assay was used together with several simple clinical parameters [8]. As a promising result, the Authors found that around half of those patients, who were later identified a sepsis cases, were already recognized in the prehospital setting, and moreover, that the final hospital mortality of the patients, in whom the algorithm was applied, was significantly lower compared with patients receiving standard management. A recent review tried to put together the literature and finally stated that (1) recognition of sepsis by ambulance clinicians is poor, (2) the use of screening tools, based on the SSC diagnostic criteria, improves prehospital sepsis recognition, and (3) screening tools derived from EMS data have been developed, but they have not yet been validated in clinical practice [9].

In conclusion, there currently is an open discussion on the ideal time point of early treatment, especially regarding administration of empiric antibiotics when no clear diagnosis is available. But, on the other hand, we must ask: what are we waiting for? Why should 3 h waiting time be better than 1 h? There will be no result from the blood cultures or from any other microbiological sample. For the prehospital setting, it must be admitted that we cannot have a clear diagnosis of sepsis as defined by current definitions since several parameters of the SOFA score are not available. Reason enough to foster clinical studies in this context!

##### **References** 

1. Machiavelli, N. *The Prince*; Peacock Books: Adelaide, Australia, 2017
2. Rhodes, A.; Evans, L.E.; Alhazzani, W.; Levy, M.M.; Antonelli, M.; Ferrer, R.; Kumar, A.; Sevransky, J.E.; Sprung, C.L.; Nunnally, M.E.; et al. Surviving Sepsis Campaign: International Guidelines for Management of Sepsis and Septic Shock: 2016. *Intensiv. Care Med.*
**2017**, *43*, 304–377.
3. Levy, M.M.; Dellinger, R.P.; Townsend, S.R.; Linde-Zwirble, W.T.; Marshall, J.C.; Bion, J.; Schorr, C.; Artigas, A.; Ramsay, G.; Beale, R.; et al. The Surviving Sepsis Campaign: results of an international guideline-based performance improvement program targeting severe sepsis. *Crit. Care Med*. **2010**, *38*, 367–374.
4. Seymour, C.W.; Gesten, F.; Prescott, H.C.; Friedrich, M.E.; Iwashyna, T.J.; Phillips, G.S.; Lemeshow, S.; Osborn, T.; Terry, K.M.; Levy, M.M. Time to Treatment and Mortality during Mandated Emergency Care for Sepsis. *N. Engl. J. Med.*
**2017**, *376*, 2235–2244.
5. Levy, M.M.; Evans, L.E.; Rhodes, A. The Surviving Sepsis Campaign Bundle: 2018 Update. *Crit. Care Med.*
**2018**, *46*, 997–1000.
6. Disselkamp, M.; Yataco, A.O.C.; Simpson, S.Q. POINT: Should Broad-Spectrum Antibiotics Be Routinely Administered to All Patients With Sepsis as Soon as Possible? Yes. *Chest*
**2019**, *156*, 645–647.
7. Patel, J.J.; Bergl, P.A. COUNTERPOINT: Should Broad-Spectrum Antibiotics Be Routinely Administered to All Patients With Sepsis as Soon as Possible? No. *Chest*
**2019**, *156*, 647–649.
8. Guerra, W.F.; Mayfield, T.R.; Meyers, M.S.; Clouatre, A.E.; Riccio, J.C. Early Detection and Treatment of Patients with Severe Sepsis by Prehospital Personnel. *J. Emerg. Med.*
**2013**, *44*, 1116–1125.
9. Smyth, M.A.; Brace-McDonnell, S.J.; Perkins, G.D. Identification of adults with sepsis in the prehospital environment: a systematic review. *BMJ Open*
**2016**, 6, e011218.

### 2.2. SESION II. FLUID THERAPY AND VASSOPRESSORS

#### **2.2.1. Use of Vasopressors in Septic Shock** 

HamzaouiOlfaICU, Beclere University Hospital, Clamart, France

In spite of multiple therapeutic advances over the past decades, septic shock remains the leading cause of morbidity and mortality in critically ill patients [1]. 

##### **Why to Use Vasopressors in Septic Shock?** 

Besides relative and absolute hypovolemia, depressed vascular tone is a major characteristic of septic shock, mostly due to excessive release of inflammatory mediators. Vascular tone depression must lead to severe hypotension, which may induce hypoperfusion of vital organs if mean arterial pressure (MAP) decreased below a certain critical level [2]. In this regard, correction of severe hypotension with vasopressors can improve regional blood flows as suggested by studies showing an improvement in urine flow [3,4] and creatinine clearance [4] with increased MAP but unchanged cardiac output.

##### **Which First-Line Vasopressor?** 

There is nowadays a consensus to recommend norepinephrine as the fist-line vasopressor in septic shock [5,6]. This recommendation is supported by a meta-analysis that clearly showed the superiority of norepinephrine over dopamine [7].

##### **When to Start the Vasopressor?** 

In the past, the vasopressor therapy was started after complete correction of hypovolemia. Nowadays, the vasopressor—namely norepinephrine—is most often initiated earlier [6]. There are several arguments that support this therapeutic strategy: 

(1) *Early initiation of norepinephrine can prevent prolonged and severe hypotension.* Severe and prolonged hypotension is associated with a poor outcome in septic shock [8]. In presence of depressed arterial tone, fluid administration alone cannot restore a sufficient MAP. Early administration of norepinephrine can thus shorten the duration of severe.

(2) *Early initiation of norepinephrine can increase cardiac output through an increase in cardiac preload.* Due to its α_1_-adrenergic properties, norepinephrine is able to redistribute blood from unstressed to stressed volume [9] and thus to increase cardiac preload. As septic patients are most often preload responsive at the early phase, norepinephrine can thus increase cardiac output when administered early [10]. 

(3) *Early initiation of norepinephrine can also increase cardiac output through an increase in cardiac contractility.* Due to its β_1_-adrenergic properties, norepinephrine is able to enhance contractility as recently demonstrated [11]. This effect should occur at the early phase of sepsis, as the β_1_-adrenergic receptors are not yet downregulated. In addition, as norepinephrine increases vascular tone, it increases the diastolic arterial pressure (DAP) and thus prevent or correct left ventricular myocardial ischemia in predisposed patients (especially those with prior coronary artery disease), what can also improve cardiac contractility.

(4) Early initiation of norepinephrine results in less fluid requirements [12] and should prevent harmful fluid overload.

(5) Early initiation of norepinephrine should eventually improve outcome as suggested by a recent randomized placebo-controlled clinical trial [13].

A simple way to identify patients who need urgent initiation of norepinephrine is the presence of a low DAP, which generally reflects a low arterial tone [14].

##### **What Is the Optimal Blood Pressure to Target?** 

There is no universally admitted MAP threshold ensuring that blood flow is independent of arterial pressure in most vital organs. Nevertheless, in septic shock, current resuscitation guidelines recommend to achieve and maintain MAP ≥ 65 mmHg, in order to avoid additional organ hypoperfusion [5,6]. Patients with prior hypertension are supposed to have a rightward shift of the autoregulation curve (organ blood flow vs. organ perfusion pressure). This should result in a higher MAP to target in this subpopulation of patients as suggested by a randomized controlled trial that showed benefits in renal function when septic shock patients with chronic hypertension are managed with a higher MAP target (80–85 mmHg vs. 65–70 mmHg) [15].

##### **What to Do in Cases of Refractory Hypertension?** 

There is no consensual definition of refractory hypertension. For some experts (but not all), it is defined by inability of 1 µg/kg/min norepinephrine to reach 65 mmHg MAP. It is noteworthy that some studies like that by Auchet et al. showed that even for doses far higher than 1 µg/kg/min, 40% of patients with septic shock can be discharged alive [16]. Nevertheless, current guidelines recommend adding vasopressin to reach the MAP target or to reduce the norepinephrine dosage. Note that recent randomized controlled trial did not find any benefits in adding vasopressin to norepinephrine [17–19] so that the question is still open. Adding Angiotensin II to norepinephrine is not recommended, although there is data showing that the former agent can reduce the dosage of the latter [20], which would theoretically result in less harmful effects of norepinephrine. Maybe the future is to combine norepinephrine, vasopressin and angiotensin II at low doses not as a rescue therapy but at the beginning of resuscitation [21]. Finally, in cases of escalating doses of norepinephrine, administration of hemisuccinate of hydrocortisone is a reasonable option [5,6] with the aim of accelerating shock resolution [22,23] and eventually improving outcome [23].

###### **References** 

1. Shankar-Hari, M.; Phillips, G.S.; Levy, M.L.; Seymour, C.W.; Liu, V.X.; Deutschman, C.S.; Angus, D.C.; Rubenfeld, G.D.; Singer, M.; Force, S.D.T. Developing a New Definition and Assessing New Clinical Criteria for Septic Shock: For the Third International Consensus Definitions for Sepsis and Septic Shock (Sepsis-3). *JAMA*
**2016**, *315*, 775–787.
2. Hamzaoui, O.; Scheeren, T.W.L.; Teboul, J.L. Norepinephrine in septic shock: when and how much? *Curr. Opin. Crit. Care*
**2017**, *23*, 342–347.
3. Desjars, P.; Pinaud, M.; Potel, G.; Tasseau, F.; Touze, M.-D. A reappraisal of norepinephrine therapy in human septic shock. *Crit. Care Med.*
**1987**, *15*, 134–137.
4. Albanèse, J.; Leone, M.; Delmas, A.; Martin, C. Terlipressin or norepinephrine in hyperdynamic septic shock: a prospective, randomized study. *Crit. Care Med.*
**2005**, *33*, 1897–1902.
5. Rhodes, A.; Evans, L.E.; Alhazzani, W.; Levy, M.M.; Antonelli, M.; Ferrer, R.; Kumar, A.; Sevransky, J.E.; Sprung, C.L.; Nunnally, M.E.; et al. Surviving Sepsis Campaign: International Guidelines for Management of Sepsis and Septic Shock: 2016. *Intensiv. Care Med.*
**2017**, *43*, 304–377.
6. Scheeren, T.W.L.; Bakker, J.; De Backer, D.; Annane, D.; Asfar, P.; Boerma, E.C.; Cecconi, M.; Dubin, A.; Dünser, M.W.; Duranteau, J.; et al. Current use of vasopressors in septic shock. *Ann. Intensiv. Care*
**2019**, *9*, 20.
7. De Backer, D.; Aldecoa, C.; Njimi, H.; Vincent, J. Dopamine versus norepinephrine in the treatment of septic shock: a meta-analysis*. *Crit. Care Med*. **2012**, *40*, 725–730.
8. Varpula, M.; Tallgren, M.; Saukkonen, K.; Voipio-Pulkki, L.-M.; Pettilä, V. Hemodynamic variables related to outcome in septic shock. *Intensiv. Care Med.*
**2005**, *31*, 1066–1071.
9. Persichini, R.; Silva, S.; Teboul, J.L.; Jozwiak, M.; Chemla, D.; Richard, C.; Monnet, X. Effects of norepinephrine on mean systemic pressure and venous return in human septic shock. *Crit. Care Med*. **2012**, *40*, 3146–3153.
10. Hamzaoui, O.; Georger, J.-F.; Monnet, X.; Ksouri, H.; Maizel, J.; Richard, C.; Teboul, J.-L. Early administration of norepinephrine increases cardiac preload and cardiac output in septic patients with life-threatening hypotension. *Crit. Care*
**2010**, *14*, R142.
11. Hamzaoui, O.; Jozwiak, M.; Geffriaud, T.; Sztrymf, B.; Prat, D.; Jacobs, F.; Monnet, X.; Trouiller, P.; Richard, C.; Teboul, J.L. Norepinephrine exerts an inotropic effect during the early phase of human septic shock. *Br. J. Anaesth*. **2018**, *120*, 517–524.
12. Bai, X.; Yu, W.; Ji, W.; Lin, Z.; Tan, S.; Duan, K.; Dong, Y.; Xu, L.; Li, N. Early versus delayed administration of norepinephrine in patients with septic shock. *Crit. Care*
**2014**, *18*, 532.
13. Permpikul, C.; Tongyoo, S.; Viarasilpa, T.; Trainarongsakul, T.; Chakorn, T.; Udompanturak, S. Early Use of Norepinephrine in Septic Shock Resuscitation (CENSER): A Randomized Trial. *Am. J. Respir. Crit. Care Med.*
**2019**, *199*, 1097–1105.
14. Hamzaoui, O.; Teboul, J.-L. Importance of diastolic arterial pressure in septic shock: PRO. *J. Crit. Care*
**2019**, *51*, 238–240.
15. Asfar, P.; Meziani, F.; Hamel, J.-F.; Grelon, F.; Mégarbane, B.; Anguel, N.; Mira, J.-P.; Dequin, P.-F.; Gergaud, S.; Weiss, N.; et al. High versus Low Blood-Pressure Target in Patients with Septic Shock. *N. Engl. J. Med.*
**2014**, *370*, 1583–1593.
16. Auchet, T.; Regnier, M.-A.; Girerd, N.; Levy, B. Outcome of patients with septic shock and high-dose vasopressor therapy. *Ann. Intensiv. Care*
**2017**, *7*, 43.
17. Gordon, A.C.; Mason, A.J.; Thirunavukkarasu, N.; et al. Effect of Early Vasopressin vs. Norepinephrine on Kidney Failure in Patients With Septic Shock: The VANISH Randomized Clinical Trial. *JAMA*
**2016**, *316*, 509–518.
18. Laterre, P.-F.; Berry, S.M.; Blemings, A.; Carlsen, J.E.; François, B.; Graves, T.; Jacobsen, K.; Lewis, R.J.; Opal, S.M.; Perner, A.; et al. Effect of Selepressin vs. Placebo on Ventilator- and Vasopressor-Free Days in Patients With Septic Shock: The SEPSIS-ACT Randomized Clinical Trial. *JAMA*
**2019**, *322*, 1476.
19. Hajjar, L.A.; Zambolim, C.; Belletti, A.; et al. Vasopressin Versus Norepinephrine for the Management of Septic Shock in Cancer Patients: The VANCS II Randomized Clinical Trial. *Crit Care Med*. **2019**, *47*, 1743–1750.
20. Khanna, A.; Ostermann, M.; Bellomo, R. Angiotensin II for the Treatment of Vasodilatory Shock. *N. Engl. J. Med.*
**2017**, *377*, 2604.
21. Teboul, J.L.; Duranteau, J.; Russell, J.A. Intensive Care Medicien in 2050: vasopressors in sepsis. *Intensive Care Med.*
**2018**, *44*, 1130–1132.
22. Venkatesh, B.; Finfer, S.; Cohen, J.; et al. Adjunctive Glucocorticoid Therapy in Patients with Septic Shock. *N. Engl. J. Med*. **2018**, *378*, 797–808.
23. Annane, D.; Renault, A.; Brun-Buisson, C.; Mégarbane, B.; Quenot, J.-P.; Siami, S.; Cariou, A.; Forceville, X.; Schwebel, C.; Martin-Loeches, I.; et al. Hydrocortisone plus Fludrocortisone for Adults with Septic Shock. *N. Engl. J. Med.*
**2018**, *378*, 809–818.

#### **2.2.2. Albumin Fluid Resuscitation in Patients with Septic Shock** 

ArtigasAntonioCritical Care Center, Corporació Sanitaria Universitaria Parc Tauli, CIBER Enfermedades Respiratorias, Autonomous University of Barcelona, Spain

Fluid administration is often the first line treatment in patients with sepsis and septic shock, but both too much and too little fluid can negatively impact on patients outcome.In cases of “fluid unresponsiveness”, fluid can only exert some deleterious effects.It is now well demonstrated that excessive fluid administration is harmful especially during sepsis and septic shock [1–4]. Then, predicting whether volume expansion will result in the expected increase in cardiac output is one of daily challenges when treating such patients.Recent studies have improved our evidence-base regarding the different fluids available, but each solution has a specific profile and choices must be guided by individual patient characteristics [5].

Colloid solutions remain longer in intravascular space thus are less likely to cause edema, and should considered in combination with crystalloids and vasopressors, especially in patients likely to require large fluid volumes and in patients with sepsis and acute lung injury [6,7]. The only natural colloid is human albumin, which may have beneficial effects in patients with sepsis. In addition to its effects on volume expansion, albumin transport sphingosin-1-phosphate which has protective endothelial effects, acts as a free radical scavenger, and has immunomodulatory and anti-inflammatory effects. Plasma protein such albumin is physiologically bound within the glicocalix, thus contributing to stability of the layer [8,9].

Low serum albumin concentration has been reported in 30% to 50% of critically ill patients associated with a higher mortality rates. Therefore, the potential benefit of albumin administration in patients with sepsis has been tested in different clinical trials (CT). Randomized CTs comparing albumin to crystalloids resuscitation fluids found no overall differences, however a consistent effect has been observed in subgroups of patients with septic shock with a decreased in mortality rate and a greater catecolamine free days [10]. Patients with septic shock and hypoalbuminemia may represent the most suitable target population [11]. Several ongoing CTs may provide additional evidence and the role of the additional physiologic effects of albumin.

##### **References** 

1. Murphy, C.V.; Schramm, G.E.; Doherty, J.A.; Reichley, R.M.; Gajic, O.; Afessa, B.; Micek, S.T.; Kollef, M.H. The importance of fluid management in acute lung injury secondary to septic shock. *Chest*
**2009**, *136*, 102–109.
2. Malbrain, M.L.N.G.; Van regenmortel, N.; Saugel, B.; Tavernier, B.; Van Gaal, P.J.; Joannes-Boyau, O.; Teboul, J.L.; Rice, T.W.; Mythen, M.; Monnet, X. Principles of fluid management and stewardship in septic shock: it is time for consider the four D´s and the four phases of fluid therapy. *Ann Intensive Care*
**2018**, *8*, 66.
3. Sakr, Y.; Rubatto Birri, P.N.; Kotfis, K.; Nanchal, R.; Sha, B.; Kluge, S.; Schroeder, M.E.; Marshall, J.C.; Vincent, J.L. Intensive care Over Nations Investigators. Higher fluid balance increases the risk of death from sepsis: results from a large international audit. *Crit. Care Med*. **2017**, *45*, 386–394.
4. Genga, K.R.; Russell, J.A. How much excess fluid impairs outcome of sepsis? *Intensive Care Med*. **2017**, *43*, 680–682.
5. Murphy, J.A.; Mythen, M.G. Resuscitation fluids. *N. Engl. J. Med*. **2013**, *369*, 1243–1251.
6. Aya, H.D.; Ster, I.C.; Fletcher, N.; Grounds, R.M.; Rhodes, A.; Cecconi, M. Pharmacodynamics analysis of fluid challenge. *Crit. Care Med*. **2016**, *44*, 880–891.
7. Nunes, T.S.; Ladeira, R.T.; Bafi, A.T.; de Azevedo, L.C.; Machado, F.R.; Freitas, F.G. Duration of hemodynamic effects of crystalloids in patients with circulatory shock after initial resuscitation. *Ann. Intensive Care*
**2014**, *4*, 25.
8. Thuy, A.V.; Reimann, C.M.; Hemdan, N.Y.; Gräler, M.H. Sphingosine 1-phosphate in blood: function, metabolism, and fate. *Cell Physiol. Biochem*. **2014**, *34*, 158–171.
9. Hariri, G.; Joffre, J.; Devyckere, S.; Bigé, N.; Dumas, G.; Baudel, J.L.; Maury, E.; Guidet, B.; Ait-Oufella, H. Albumin improves endothelial function in septic shock patients: a pilot study. *Intensive Care Med*. **2018**, *44*, 669–671.
10. Carioni, P.; Tognoni, G.; Masson, S.; Fumagalli, R.; Pesenti, A.; Romero, M.; Fanizza, C.; Casponi, L.; Faenza, S.; Grasselli, G.; et al. ALBIOS Study Investigators. Albumin replacement in patients with severe sepsis or septic shock. *N. Engl. J. Med*. **2014**, *370*, 1412–1421.
11. Vincent, J.L.; Russell, J.A.; Jacob, M.; Martin, G.; Guidet, B.; Wenerman, J.; Ferrer, R.; McClustey, S.A.; Gattinoni, L. Albumin administration in acutely ill: what is new and where next? *Crit. Care*
**2014**, *18*, 231.

#### **2.2.3. Arguments for an Early Administration of Norepinephrine in Septic Shock** 

TeboulJean-LouisMedical ICU, Bicetre University Hospital, Paris-Saclay University, Le Kremlin-Bicêtre, France

##### **Introduction** 

Decreased vascular tone is a hallmark of septic shock and contributes much to the severity of sepsis-induced hypotension. Physiologically, organ blood flow depends on perfusion pressure when mean arterial pressure decreases below a certain critical value. It is thus important to correct severe hypotension as early as possible and not to delay the initiation of vasopressors, even before completion of fluid resuscitation [1]. Norepinephrine is recommended as the first-line vasopressor in septic shock [2]. There are many important arguments that support the recommendation from a group of 34 experts—all members of the European Society of Intensive Care Medicine [1]—in favor of early administration of norepinephrine in septic shock. 

##### **Arguments Supporting Early Initiation of Norepinephrine** 

*(1). Early initiation of norepinephrine can prevent prolonged and severe hypotension*

Not only the degree but also the duration of hypotension is associated with poor outcome in septic shock [3]. In presence of depressed arterial tone, fluid administration alone is not enough to correct severe hypotension and can even potentially decrease vascular tone [4]. Early initiation of a powerful vasopressor such as norepinephrine—in combination with fluid therapy—can thus shorten the duration of hypotension in comparison with fluid alone. 

*(2). Early initiation of norepinephrine increases cardiac output in septic shock*

Many studies showed that norepinephrine increases cardiac output when initiated early in patients with septic shock [5]. In a study that included 105 severely hypotensive patients, the increase in cardiac output with norepinephrine was associated with an increase in cardiac preload and a reduction in pulse pressure variation [6]. Such results suggest that norepinephrine through its α_1_-adrenergic effects is able to redistribute venous blood from the unstressed to the stressed blood volume as confirmed by a study that reported an increase in mean systemic filling pressure with norepinephrine [7]. Since septic patients are most often preload responsive at the early phase, the increase in cardiac preload can thus increase cardiac output. Moreover, norepinephrine through β_1_-adrenergic stimulation may increase left and right ventricular contractility when administered early as suggested by results of a recent clinical study where norepinephrine was initiated in the first three hours of resuscitation [8]. 

*(3). Early initiation of norepinephrine may improve microcirculation in septic shock*

In severely hypotensive patients, restoration of organ perfusion pressure with norepinephrine may result in improved microcirculatory blood flow as suggested by a study using near-infrared spectroscopy at the level of the thenar eminence in patients with septic shock [9]. 

*(4). Early initiation of norepinephrine prevents harmful fluid overload*

Positive fluid balance is independently associated with increased mortality in septic shock [10]. As norepinephrine also increases cardiac preload and cardiac output, its early initiation should reduce the volume of infused fluids. In a retrospective study in patients with septic shock, patients in whom norepinephrine was started within the first two hours of resuscitation received fewer fluids than those who received a delayed norepinephrine administration [11]. 

*(5). Early initiation of norepinephrine might improve outcome*

In a cohort of 213 patients with septic shock, the time to initiate norepinephrine was reported to be an independent factor associated with mortality [11]. Interestingly, in the subgroup of patients who received norepinephrine within the first two hours of resuscitation, the duration of administration of norepinephrine was shorter while the total dose of norepinephrine was lower than in the subgroup of patients in whom initiation of norepinephrine was delayed [11]. Recently, a randomized controlled trial compared two strategies of norepinephrine initiation: early initiation (n = 155) vs. delayed initiation (n = 155). The primary outcome was shock control rate by six hours after diagnosis. The shock control was defined as: (1) mean arterial pressure of at least 65 mmHg and (2) either urine output ≥0.5 mL/kg/h for 2 consecutive hours or a decrease in lactate ≥10% from baseline. Shock was controlled in significantly more patients in the early norepinephrine initiation arm compared to the delayed norepinephrine initiation arm [12]. In addition, there were lower rates of cardiogenic pulmonary edema and of new episodes of cardiac arrhythmia in the early vs. delayed norepinephrine initiation. These findings clearly support the benefit of early initiation of norepinephrine in septic shock. 

##### **Identification of Patients Who Need Urgent Initiation of Norepinephrine** 

In patients with sepsis-induced hypotension, norepinephrine should be initiated when the vascular tone depression is judged to contribute significantly to hypotension. As the diastolic arterial blood pressure (DAP) reflects the vascular tone, a low DAP (e.g., <40 mmHg) is strongly suggestive of a markedly depressed arterial tone and should prompt initiation of norepinephrine urgently [13].

##### **Conclusions** 

Early administration of norepinephrine during septic shock may benefit to patients through an increase in cardiac output, due to an increase in cardiac preload and in cardiac contractility, an improvement of microcirculation and a limitation of fluid overload. Recent data suggest that early administration of norepinephrine may improve outcome. In the case of any doubt, the clinician should look at the DAP, a marker of vascular tone. A low DAP can simply identify patients who need norepinephrine urgently. 

###### **References** 

1. Scheeren, T.W.L.; Bakker, J.; De Backer, D.; Annane, D.; Asfar, P.; Boerma, E.C.; Cecconi, M.; Dübin, A.; Dünser, M.W.; Duranteau, J.; et al. Current use of vasopressors in septic shock. *Ann. Intensive Care*
**2019**, *9*, 20.
2. Rhodes, A.; Evans, L.E.; Alhazzani, W.; Levy, M.M.; Antonelli, M.; Ferrer, R.; Kumar, A.; Sevransky, J.E.; Sprung, C.L.; Nunnaly, M.E.; et al. Surviving Sepsis Campaign: International Guidelines for Management of Sepsis and Septic Shock: 2016. *Intensive Care Med*. **2017**, *45*, 486–552.
3. Varpula, M.; Tallgren, M.; Saukkonen, K.; Voipio-Pulkki, L.M.; Pettilä, V. Hemodynamic variables related to outcome in septic shock. *Intensive Care Med*. **2005**, *31*, 1066–1071.
4. Pierrakos, C.; Velissaris, D.; Scolletta, S.; Heenen, S.; De Backer, D.; Vincent, J.L. Can changes in arterial pressure be used to detect changes in cardiac index during fluid challenge in patients with septic shock? *Intensive Care Med*. **2012**, *38*, 422–428.
5. Hamzaoui, O.; Scheeren, T.W.L.; Teboul, J.L. Norepinephrine in septic shock: when and how much? *Curr. Opin. Crit. Care*
**2017**, *23*, 342–347.
6. Hamzaoui, O.; Georger, J.F.; Monnet, X.; Ksouri, H.; Maizel, J.; Richard, C.; Teboul, J.L. Early administration of norepinephrine increases cardiac preload and cardiac output in septic patients with life-threatening hypotension. *Crit. Care*
**2010**, *14*, R142.
7. Persichini, R.; Silva, S.; Teboul, J.L.; Jozwiak, M.; Chemla, D.; Richard, C.; Monnet, X. Effects of norepinephrine on mean systemic pressure and venous return in human septic shock. *Crit. Care Med*. **2012**, *40*, 3146–3153.
8. Hamzaoui, O.; Jozwiak, M.; Geffriaud, T.; Sztrymf, B.; Prat, D.; Jacobs, F.; Monnet, X.; Trouiller, P.; Richard, C.; Teboul, J.L. Norepinephrine exerts an inotropic effect during the early phase of human septic shock. *Br. J. Anaesth*. **2018**, *120*, 517–524.
9. Georger, J.F.; Hamzaoui, O.; Chaari, A.; Maizel, J.; Richard, C.; Teboil, J.L. Restoring arterial pressure with norepinephrine improves muscle tissue oxygenation assessed by near-infrared spectroscopy in severely hypotensive septic patients. *Intensive Care Med*. **2010**, *36*, 1882–1889.
10. Sakr, Y.; Rubatto Birri, P.N.; Kotfis, K.; Nanchal, R.; Shah, B.; Kluge, S.; Schroeder, M.E.; Marshall, J.C.; Vincent, J.L.; Intensive Care Over Nations Investigators. Higher Fluid Balance Increases the Risk of Death From Sepsis: Results From a Large International Audit. *Crit. Care Med*. **2017**, *45*, 386–394.
11. Bai, X.; Yu, W.; Ji, W.; Lin, Z.; Tan, S.; Duan, K.; Dong, Y.; Xu, L.; li, N. Early versus delayed administration of norepinephrine in patients with septic shock. *Crit. Care*. **2014**, *18*, 532.
12. Permpikul, C.; Tongyoo, S.; Viarasilpa, T.; Trainarongsakul, T.; Chakorn, T.; Udompanturak, S. Early Use of Norepinephrine in Septic Shock Resuscitation (CENSER). A Randomized Trial. *Am. J. Respir. Crit Care Med*. **2019**, *199*, 1097–1105.
13. Hamzaoui, O.; Teboul, J.L. Importance of diastolic arterial pressure in septic shock: PRO. *J. Crit. Care*
**2019**, *51*, 238–240.

#### **2.2.4. Early vs. Delayed Norepinephrine Administration in Septic Shock? No** 

De BackerDanielDepartment of Intensive Care, CHIREC Hospitals, Université Libre de Bruxelles, Brussels, Belgium

Patients with septic shock require the combined administration of fluids and vasopressors. Septic shock is defined as “a subset of sepsis in which profound circulatory, cellular and metabolic abnormalities are associated with a greater risk of mortality than in sepsis alone” (1). It is best identified as “hypotension requiring use of vasopressors to maintain a mean blood pressure of 65 mmHg or greater and having a serum lactate level greater than 2 mmol/L persisting after adequate fluid resuscitation” (2). Apparently, this may imply that shock require vasopressor *per definition*, so that the debate would be useless. However other definitions importantly state that shock is a life-threatening form of acute circulatory failure associated with inadequate oxygen utilization, a condition in which hypotension is not a prerequisite (3). Hence the definitions highlight the role of tissue perfusion. Blood pressure, and thus perfusion pressure, is only one of determinants tissue perfusion, cardiac output, regional blood flow distribution and microcirculatory perfusion are the other key determinants of tissue perfusion.

When discussing early versus delayed norepinephrine administration, we are thus discussing the balance between vasoconstriction preserving tissue perfusion and flow.

To which extend hypotension can be tolerated and for how long? Observational data suggest that the more severe the hypotension, the shortest is the time that can be safely spent at this level of pressure (4). Some observational studies also suggest that the delay between onset of hypotension and initiation of vasopressors may be associated with increased mortality (5).

While these observational studies provide important information associating hypotension and outcome, there is no proof of a causative association. Severity of disease may indeed be associated with more severe hypotension and more difficulties to correct hypotension, hence a prolonged time spent with hypotension despite efforts made to correct hypotension. At this stage, there is no proof that rapid correction of hypotension by early initiation of vasopressors may be associated with better outcome compared to delayed initiation of vasopressors while making important efforts to increase organ perfusion.

Preserving tissue perfusion is often better obtained with fluid and inotrope administration than with vasopressors. In many trials evaluating the potential effect of resuscitation strategies, optimization of tissue perfusion was achieved using initially more fluids and more dobutamine, while vasopressor doses were identical (6). Vasopressor also have variable effects on the microcirculation (7, 8), with paradoxical impairment of microcirculatory perfusion at high norepinephrine doses in some patients (8). On the other hand, fluids improve microvascular perfusion at the early stages of sepsis (9). In addition, vasodilatory agents may sometimes improve microcirculatory perfusion in patients with septic shock (10, 11), after adequate volume resuscitation.

In addition, vasopressor agents are associated with significant adverse effects that can also increase the risk of death. Observational studies have shown that the higher the vasopressor load, the higher the mortality (12). In a trial testing higher mean arterial pressure target, there was no difference in outcome at the higher blood pressure target, but the higher pressure target was requiring higher doses of vasopressors which resulted in a higher incidence of atrial fibrillation (13). Hence, minimizing vasopressor doses may be a strategy in itself.

On the other hand, there are even small series of septic shock suggesting that hypotension, sometimes severe, can be tolerated without sequalae as long as tissue perfusion is preserved (14).

Accordingly, even though observational data suggest that the severity and duration of hypotension are associated with worse outcome, there are no data at this stage supporting the early use of vasopressors, before optimization of tissue perfusion. As shock is characterized by an inadequate tissue perfusion, optimizing tissue perfusion should be the first target of our resuscitation efforts, and restauration of perfusion pressure is only one part of this strategy that should not prevail the other efforts that are usually more efficient.

##### **References** 

1. Singer, M.; Deutschman, C.S.; Seymour, C.W.; Shankar-Hari, M.; Annane, D.; Bauer, M.; Bellomo, R.; Bernard, G.R.; Chiche, J.-D.; Coopersmith, C.M.; et al. The Third International Consensus Definitions for Sepsis and Septic Shock (Sepsis-3). *JAMA*
**2016**, *315*, 801–10.
2. Shankar-Hari, M.; Phillips, G.S.; Levy, M.L.; Seymour, C.W.; Liu, V.X.; Deutschman, C.S.; Angus, D.C.; Rubenfeld, G.D.; Singer, M.; Force, S.D.T. Developing a New Definition and Assessing New Clinical Criteria for Septic Shock: For the Third International Consensus Definitions for Sepsis and Septic Shock (Sepsis-3). *JAMA*
**2016**, *315*, 775–87.
3. Cecconi, M.; De Backer, D.; Antonelli, M.; Beale, R.; Bakker, J.; Hofer, C.; Jaeschke, R.; Mebazaa, A.; Pinsky, M.R.; Teboul, J.L.; et al. Consensus on circulatory shock and hemodynamic monitoring. Task force of the European Society of Intensive Care Medicine. *Intensiv. Care Med.*
**2014**, *40*, 1795–815.
4. Vincent, J.-L.; Nielsen, N.D.; Shapiro, N.I.; Gerbasi, M.E.; Grossman, A.; Doroff, R.; Zeng, F.; Young, P.J.; Russell, J.A. Mean arterial pressure and mortality in patients with distributive shock: a retrospective analysis of the MIMIC-III database. *Ann. Intensiv. Care*
**2018**, *8*, 107.
5. Bai, X.; Yu, W.; Ji, W.; Lin, Z.; Tan, S.; Duan, K.; Dong, Y.; Xu, L.; Li, N. Early versus delayed administration of norepinephrine in patients with septic shock. *Crit. Care*
**2014**, *18*, 532.
6. Rivers, E.; Nguyen, B.; Havstad, S.; Ressler, J.; Muzzin, A.; Knoblich, B.; Peterson, E.; Tomlanovich, M. Early Goal-Directed Therapy in the Treatment of Severe Sepsis and Septic Shock. *New Engl. J. Med.*
**2001**, *345*, 1368–1377.
7. Thooft, A.; Favory, R.; Salgado, D.R.; Taccone, F.S.; Donadello, K.; De Backer, D.; Creteur, J.; Vincent, J.-L. Effects of changes in arterial pressure on organ perfusion during septic shock. *Crit. Care*
**2011**, *15*, R222.
8. Dubin, A.; O Pozo, M.; A Casabella, C.; Pálizas, F.; Murias, G.; Moseinco, M.C.; Edul, V.S.K.; Estenssoro, E.; Ince, C.; Pálizas, F. Increasing arterial blood pressure with norepinephrine does not improve microcirculatory blood flow: a prospective study. *Crit. Care*
**2009**, *13*, R92.
9. Ospina-Tascon, G.; Neves, A.P.; Occhipinti, G.; Donadello, K.; Büchele, G.; Simion, D.; Chierego, M.-L.; Silva, T.O.; Fonseca, A.; Vincent, J.-L.; et al. Effects of fluids on microvascular perfusion in patients with severe sepsis. *Intensiv. Care Med.*
**2010**, *36*, 949–955.
10. E Spronk, P.; Ince, C.; Gardien, M.J.; Mathura, K.R.; Straaten, H.M.O.-V.; Zandstra, D.F. Nitroglycerin in septic shock after intravascular volume resuscitation. *Lancet*
**2002**, *360*, 1395–1396.
11. Depret F, Sitbon A, Soussi S, De Tymowski C, Blet A, Fratani A, et al. Intravenous iloprost to recruit the microcirculation in septic shock patients? *Intensive Care Med*. **2018**, *44*, 121–122.
12. Dünser, M.W.; Ruokonen, E.; Pettilä, V.; Ulmer, H.; Torgersen, C.; A Schmittinger, C.; Jakob, S.; Takala, J. Association of arterial blood pressure and vasopressor load with septic shock mortality: a post hoc analysis of a multicenter trial. *Crit. Care*
**2009**, *13*, R181.
13. Asfar, P.; Meziani, F.; Hamel, J.-F.; Grelon, F.; Mégarbane, B.; Anguel, N.; Mira, J.-P.; Dequin, P.-F.; Gergaud, S.; Weiss, N.; et al. High versus Low Blood-Pressure Target in Patients with Septic Shock. *New Engl. J. Med.*
**2014**, *370*, 1583–1593.
14. Lavillegrand, J.-R.; Dumas, G.; Bigé, N.; Zafimahazo, D.; Guidet, B.; Maury, E.; Ait-Oufella, H. Should we treat mild hypotension in septic patients in the absence of peripheral tissue hypoperfusion? *Intensiv. Care Med.*
**2018**, *44*, 1593–1594.

### 2.3. SESION III. Personalize Sepsis Care

#### **2.3.1. Is Sepsis So Heterogeneous?** 

VincentJean-LouisDept of Intensive care, Erasme Hospital, Université libre de Bruxelles, Brussels, Belgium

Sepsis is defined as a dysregulated host response to infection, which results in organ failure. Although patients with sepsis may often present with similar signs of sepsis, e.g., fever, tachycardia, tachypnea, raised white blood cell count,…, sepsis is in fact a hugely heterogeneous condition, in terms of the patient (age, sex, comorbid conditions, current treatments), the underlying infection (causative microorganism, source of infection, antimicrobial susceptibilities), the degree and components of the immune response, and the severity and numbers of organs affected. Indeed, sepsis is a syndrome, not a single disease entity and characterizing the different aspects of sepsis heterogeneity is complex. However, although patient, infectious and organ failure factors all increase heterogeneity and should be taken into account when managing patients with sepsis, e.g., when considering which fluids to give, whether or not to transfuse, etc., perhaps the most difficult aspect of heterogeneity to assess and characterize is that related to the immune response. Indeed, the immune response varies between patients and in the same patient during the course of their sepsis process. One of the main reasons behind the apparent failure of anti-sepsis therapies in clinical trials is that the trials have included poorly-characterized, heterogeneous populations of patients with sepsis, particularly in terms of their immune status. Some of these patients will have responded to the intervention in question, but others will not, making the overall trial result neutral or negative. 

Rather than grouping all sepsis patients as the same and treating them all using one fixed protocol or one immunomodulatory agent, patients need to be managed as individuals. Inherent to the heterogeneity of sepsis is the understanding that there will never be a magic bullet therapy that will be effective in all patients with sepsis. Using complex analysis of large amounts of clinical data, studies have begun to identify patients with different sepsis phenotypes who may respond differently to different treatments. ’Omics approaches are also enabling identification of subsets of patients with sepsis who could benefit from different therapeutic approaches and interventions. Better characterization of sepsis phenotypes should also enable clinical trials of sepsis interventions to enroll more homogeneous populations of patients most likely to derive benefit. Improved understanding of the heterogeneity of sepsis and a greater ability to characterize it will help us treat our patients more effectively and improve patient outcomes.

Suggested reading:

- Singer M, Deutschman CS, Seymour CW et al. (2016) The Third International Consensus Definitions for Sepsis and Septic Shock (Sepsis-3). JAMA 315:801–810
- Zhang Z, Zhang G, Goyal H et al. (2018) Identification of subclasses of sepsis that showed different clinical outcomes and responses to amount of fluid resuscitation: a latent profile analysis. Crit Care. 22:347.
- Leligdowicz A, Michael A. Matthay (2019) Heterogeneity in sepsis: new biological evidence with clinical Applications. Crit Care 23:80
- Davenport EE, Burnham KL, Radhakrishnan J, et al. (2016) Genomic landscape of the individual host response and outcomes in sepsis: a prospective cohort study. Lancet Respir Med. 2016;4:259–71.
- Vincent JL (2018) The coming era of precision medicine for intensive care. Crit Care 21 (Suppl 3):314

#### **2.3.2. Rational for Probiotic Therapies as Infection Prevention Measures** 

SchultzMarcus J^1^,^2^,^3^DondorpArjen^2^,^3^1Amsterdam University Medical Centers, location ‘Academic Medical Center’, Amsterdam, The Netherlands2Department of Intensive Care & Laboratory of Experimental Intensive Care and Anaesthesiology (L·E·I·C·A); Mahidol University, Bangkok, Thailand3Mahidol–Oxford Tropical Medicine Research Unit (MORU); Department of Medicine, University of Oxford, Oxford, UK: Nuffield

##### **Background** 

ICUs serve as reservoirs of healthcare-associated pathogens that rapidly colonise the oropharynx and intestines of critically ill patients. Broad scale use of antibiotics in ICUs further induces substantial perturbations in patients’ microbial ecology resulting in overgrowth of opportunistic pathogens that could cause ventilator-associated pneumonia (VAP) if aspirated, and other hospital-acquired infections (HAIs). Studies in resource-rich ICUs have shown a beneficial effect of so-called ‘selective decontamination’ strategies with oral and enteral application of non-absorbable antibiotics throughout admission in the ICU, probably through the promotion of ‘colonisation resistance’ against opportunistic healthcare-associated microorganisms. A number of randomised trials from the Netherlands convincingly show that implementation of so-called ‘selective decontamination of the digestive tract’ (SDD) reduces ICU mortality and VAP. In many countries, however, these strategies raise concerns since the pre-existing digestive tract flora of patients on admission often contains multidrug resistant microorganisms. This includes increasing levels of resistance against antibiotics used in the above-mentioned SDD strategies, such as colistin.

##### **Probiotics** 

Probiotics are live microorganisms that promote colonisation resistance when administered in adequate quantities. Probiotic therapy has the potential to reduce the load of pathogenic bacteria, and improve survival through prevention of HAIs and VAP. The results of one recent meta-analysis of 30 trials (2972 patients) suggest beneficial effects of probiotic therapy in ICU patients (1). Probiotic therapy was associated with a significant reduction in HAIs (RR 0.80 [0.68–0.95], P = 0.009; *I*^2^* *= 36%) and VAP (RR 0.74 [0.61–0.90], P = 0.002; *I*^2^* *= 19%). Subgroup analysis indicated that the greatest improvement in the outcome of infections was in studies with a high mortality rate (RR 0.74 [0.57–0.96]; *I^2^ * = 58%, P = 0.01) than in studies with a low mortality rate (RR 0.85 [0.66–1.11]; *I^2^ * = 23%, P = 0.27). Another meta-analysis with trial sequential analysis (TSA) confirmed the finding of the abovementioned meta-analysis that probiotics could reduce the incidence of VAP in mechanically ventilated ICU patients (2). The TSA result showed that 1969 (62.9%) of the required information size of 3132 patients was accrued. The cumulative z-curve crossed the conventional boundary for benefit and crossed the trial sequential monitoring boundary for benefit, indicating that firm evidence of probiotics for preventing VAP was obtained. One large trial of probiotic therapy in ICUs (the ‘PROSPECT’ study) is currently running in Canada, USA and Saudi Arabia (Clinicaltrials.gov, NCT02462590). Results of this trial are not expected before end of 2020. A large study in Indian neonates showed that probiotic therapy prevents neonatal sepsis and death (3). A recent Japanese single centre study randomising individual patients with sepsis admitted to ICU showed a beneficial effect of probiotic therapy on the incidence of VAP and diarrhoea during ICU admission (4).

##### **Conclusions** 

Taken together, the evidence thus far shows that probiotic therapy is a very promising intervention to prevent infections, in particular HAIs and VAP, warranting further evaluation.

###### **References** 

1. Manzanares, W.; Lemieux, M.; Langlois, P.L.; Wischmeyer, P.E. Probiotic and synbiotic therapy in critical illness: a systematic review and meta-analysis. *Crit. Care*
**2016**, *19*, 262.
2. Weng, H.; Li, J.-G.; Mao, Z.; Feng, Y.; Wang, C.-Y.; Ren, X.-Q.; Zeng, X.-T. Probiotics for Preventing Ventilator-Associated Pneumonia in Mechanically Ventilated Patients: A Meta-Analysis with Trial Sequential Analysis. *Front. Pharmacol.*
**2017**, *8*.
3. Panigrahi P, Parida S, Nanda NC, Satpathy R, Pradhan L, Chandel DS, et al. A randomized synbiotic trial to prevent sepsis among infants in rural India. *Nature*
**2017**, *548*, 407–412.
4. Shimizu, K.; Yamada, T.; Ogura, H.; Mohri, T.; Kiguchi, T.; Fujimi, S.; Asahara, T.; Yamada, T.; Ojima, M.; Ikeda, M.; et al. Synbiotics modulate gut microbiota and reduce enteritis and ventilator-associated pneumonia in patients with sepsis: a randomized controlled trial. *Crit. Care*
**2018**, *22*, 239.

#### **2.3.3. CRP and Albumin Kinetics in Community-Acquired Bloodstream Infections** 

PóvoaPedro^1^,^2^,^3^1Polyvalent Intensive Care Unit, São Francisco Xavier Hospital, CHLO, Lisbon, Portugal2Nova Medical School, Faculty of Medical Sciences, CHRC, New University of Lisbon, Lisbon, Portugal3Center for Clinical Epidemiology and Research Unit of Clinical Epidemiology, OUH Odense University Hospital, Odense, Denmark

The unique personal identifier given to all residents in Scandinavian countries enables merging between administrative health registries, in which the main advantages include the reporting of population-based results and high statistical precision due to a large number of patients. Besides, the Danish public health system is tax financed and free of charge for all residents.

Community-acquired bloodstream infection (CA-BSI) is a well-defined infectious clinical entity that is associated with high morbidity and mortality. We have previously reported the prognostic prediction of one-time levels of C-reactive protein (CRP) and plasma albumin (PA) in patients with first-time CA-BSI from a geographically well-defined Danish region. In the same cohort, we recently found that the levels of CRP and PA were inversely correlated before, during and after the CA-BSI episode, even after adjustment for other clinically important cofounders, such as sepsis criteria or organ dysfunctions. With the present analysis our aim is to study CRP and PA kinetics around a CA-BSI diagnosis.

The ideal study cohort should have the same number of biochemical specimens with equal time intervals between these (as in well-designed prospective studies). This is recognized as a limitation of observational studies that present different sample numbers and time intervals between the patients. However, few, if any, studies have assessed whether this has an impact on biomarker trajectories. We therefore evaluated the impact of the number of specimens on CRP and PA trajectories around the diagnosis of CA-BSI. We studied data from 2418 CA-BSI patients, evaluating CRP and PA specimens from 30 days before through 30 days after the diagnosis of CA-BSI (D0). We divided our cohort in four subgroups based on whether specimens were occurring or not in days -30/-1, 0, 1/7 or 8/30. We showed that the mean CRP rose on day -5 and reached its peak on day 1 and the mean PA remained unchanged between day −30/0 and day 1 where it declined abruptly, followed by a slow increase thereafter. Importantly, CRP and PA trajectories around the CA-BSI episode did not differ between the four subgroups. In other words, they were independent of the number of specimens as well as the time intervals between specimens. These findings enable us to perform longitudinal analyses of real-life data in population-based studies.

Early changes in biomarker levels probably occur sometime before the BSI is diagnosed. It has clinical relevance to understand when the host inflammatory response begins. So far, this issue has not been fully addressed, particularly in community-acquired infections. Our next study was to evaluate the kinetics of CRP and PA in the 30 days before the CA-BSI. From our population-based CA-BSI database we identified 658 patients with at least one measurement of CRP or PA from day −30 (D−30) through day −1 (D−1) before D0 and a measurement of the same biomarker at D0 or D1. We found that the CRP and PA concentrations began to change inversely some days before D0, CRP increasing by day −3.1 and PA decreasing by day −1.3. From D−30 to D−4, the CRP kinetics (expressed as slopes) was −1.5 mg/L/day. From D−3 to D1, the CRP slope increased to 36.3 mg/L/day. For albumin, the slope between D−30 to D−2 was 0.1 g/L/day and changed to −1.8 g/L/day between D−1 and D1. We thus clearly showed that the biomarker levels, used as surrogates of the host inflammatory response, began to change some days before the CA-BSI diagnosis, CRP 3.1 days and PA 1.3 days before.

The last analysis aimed to evaluate the CRP and PA kinetics in response to antibiotic therapy as a predictor of short- and long-term mortality (D4-D30 and D31-D365, respectively). From the same population-based CA-BSI database we identified 935 patients with CRP and PA measurements on day 1 (D1) and D4 to evaluate the relative CRP variations in relation to the CRP value on D1 (CRP-ratio). The patients were classified according to the CRP-ratio pattern of the response previously published as fast response, slow response, non-response, and biphasic response. At D4, the CRP-ratio was lower in the survivors on D365 in comparison with D4-D30 non-survivors and D30-D365 non-survivors (*p* < 0.001). In comparison with patients alive on D4 with a fast response, those with non-response and a biphasic response had a 2.74 and 5.29 increased risk of death in D4-D30, respectively, and a 2.77 and 3.16 increased risk of death in D31-D365, respectively. The PA levels remained roughly unchanged from D1-D4, but a lower PA predicted a higher short- and long-term mortality (*p* < 0.001). The ability of the CRP-ratio and D1 PA to identify patients with a poor short- and long-term mortality after adjustments presented an acceptable discriminative performance (AUROC = 0.79). In this analysis, we showed that serial CRP measurements after CA-BSI diagnosis are clinically useful as early as D4 to identify patients with a poor outcome. Besides, the identification of individual patterns of CRP-ratio response further refines our ability to prognosticate either short- or long-term mortality. The addition of the D1 PA further increases this ability. And again, we validate the concept of the CRP-ratio pattern of response to antibiotics, as found previously in another cohort of patients (Povoa P et al. Eur Respir J 2005;25:804–812).

In conclusion, we showed that CRP and PA kinetics around a CA-BSI diagnosis could be useful in the early identification of patients that will develop a CA-BSI, and in addition, the assessment of the rate of the CRP-ratio change in response to antibiotic therapy, as well as the identification of the pattern of response, are well correlated with early and late mortality.

##### **References** 

1. Schmidt, M.; Pedersen, L.; Sørensen, H.T. The Danish Civil Registration System as a tool in epidemiology. *Eur. J. Epidemiol*. **2014**, *29*, 541–549.
2. Gradel, K.O.; Jensen, T.G.; Kolmos, H.J.; Pedersen, C.; Vinholt, P.; Lassen, A.T. Does C-reactive protein independently predict mortality in adult community-acquired bacteremia patients with known sepsis severity? *APMIS*
**2013**, *121*, 835–842.
3. Knudtzen FC et al. *J. Infect*. **2014**, *68*, 149–155.
4. Magnussen, B.; Oren Gradel, K.; Gorm Jensen, T.; Kolmos, H.J.; Pedersen, C.; Just Vinholt, P.; Touborg Lassen, A. Association between Hypoalbuminaemia and Mortality in Patients with Community-Acquired Bacteraemia Is Primarily Related to Acute Disorders. *PLoS ONE*
**2016**, *11*, e0160466.
5. Gradel, K.O.; Vinholt, P.J.; Magnussen, B.; Pedersen, C.; Jensen, T.G.; Kolmos, H.J.; Lassen, A. Hypoalbuminaemia as a marker of trans-capillary leakage in community-acquired bacteraemia patients. *Epidemiol. Infect*. **2018**, *146*, 648–655.
6. Gradel, K.O.; Póvoa, P.; Vinholt, P.J.; Magnussen, B.; Pedersen, C.; Jensen, T.G.; Kolmos, H.J.; Lassen, A.T. Real-life data patterns of C-reactive protein and albumin level trajectories around bacteremia. *Biomark. Med*. **2018**, *12*, 1251–1259.
7. Garvik, O.S. et al. *ICMx*
**2019**, *7*, (Suppl 3):000132.
8. Povoa, P.; Coelho, L.; Almeida, E.; Fernandes, A.; Mealha, R.; Moreira, P.; Sabino, H. v C-reactive protein as a marker of ventilator-associated pneumonia resolution: a pilot study. *Eur. Respir. J*. **2005**, *25*, 804–812.

### 2.4. SESION IV. New Therapies in Sepsis

#### **2.4.1. Immunological Profiling in Sepsis** 

Bermejo-MartinJesús F.^1^,^2^1Group for Biomedical Research in Sepsis (BioSepsis), Instituto de Investigación Biomédica de Salamanca (IBSAL), Paseo de San Vicente, 58–182, 37007 Salamanca, Spain; jfbermejo@saludcastillayleon.es2Hospital Universitario Río Hortega, Calle Dulzaina, 2, 47012 Valladolid, Spain

The primary cause of sepsis is an infection. Infection results from the inability of the host immunity to prevent invasion by a microbe. Sepsis is the most extreme consequence of an infection, when this causes organ failure. While the microbial side is extensively studied during the clinical management of sepsis patients, the immunological side is not. Interestingly, profiling host immune status and responses during sepsis has much to offer [1]. Both in sepsis [2] and severe pneumonia [3], there have been described a number of immunological phenotypes that are able either to predict sepsis development, to contribute to the early diagnosis of sepsis, and to identify individuals at risk of poor prognosis. We need to make worth these phenotypes to prevent and better treat sepsis patients. Lymphopenia increase risk of sepsis when it is present in individuals at the community [4], but also is an indicator of sepsis when it is established. Low expression levels of HLA-DR on monocytes identifies those sepsis patients at risk of developing secondary infections. Low levels of endogenous immunoglobulins in sepsis patients with moderate severity predict mortality [5]. Fail to expand neutrophil counts in blood is a sign of poor prognosis in septic shock [6]. Patients severe influenza [7] or sepsis [8] with transcriptomic signatures denoting adaptive immunity suppression are at higher risk of mortality. Profiling these phenotypes in sepsis patients could help to develop predictive enrichment strategies in order to identify those patients who potentially will respond better to immunotherapy. To this regard, finding out if a patient present with predominant signatures of inflammation or immunosuppression or both, will help to elucidate if he/she deserve drugs attenuating inflammation or boosting immunity, or a combination. Finally, the role of immunocompetence status in deciding antibiotic discontinuation has been neglected [9]. Does the antibiotics have the same opportunities to control infection in a patient with a severe depression of his/her immunity? The emergence of point of care devices such as Accelix TM to automatically profile HLA-DR on the surface of monocytes or Leuko TM to profile leukocyte counts in a non invasive manner, or the Nexus IB10 TM technology to profile biomarkers at the bedside od the patient will contribute to make reality the promise of immunological monitoring in sepsis. Finally, combined profiling of both endothelial and immunological markers could help to early detect the presence of organ failure during an infection [10]. 

##### **References** 

1. Rubio, I.; Osuchowski, M.F.; Shankar-Hari, M.; Skirecki, T.; Winkler, M.S.; Lachmann, G.; La Rosée, P.; Monneret, G.; Venet, F.; Bauer, M.; et al. Current gaps in sepsis immunology: new opportunities for translational research. *Lancet Infect Dis*
**2019**.
2. Bermejo-Martin, J.F.; Andaluz-Ojeda, D.; Almansa, R.; Gandía, F.; Gómez-Herreras, J.I.; Gomez-Sanchez, E.; Heredia-Rodríguez, M.; Eiros, J.M.; Kelvin, D.J.; Tamayo, E. Defining immunological dysfunction in sepsis: A requisite tool for precision medicine. *J. Infect.*
**2016**, *72*, 525–536.
3. Bermejo-Martin, J.F.; Almansa, R.; Martin-Fernandez, M.; Menendez, R.; Torres, A. Immunological profiling to assess disease severity and prognosis in community-acquired pneumonia. *Lancet Respir Med*
**2017**, *5*, e35–e36.
4. Warny, M.; Helby, J.; Nordestgaard, B.G.; Birgens, H.; Bojesen, S.E. Lymphopenia and risk of infection and infection-related death in 98,344 individuals from a prospective Danish population-based study. *PLoS Med.*
**2018**, *15*, e1002685.
5. Martin-Loeches, I.; Muriel-Bombín, A.; Ferrer, R.; Artigas, A.; Sole-Violan, J.; Lorente, L.; Andaluz-Ojeda, D.; Prina-Mello, A.; Herrán-Monge, R.; Suberviola, B.; et al. The protective association of endogenous immunoglobulins against sepsis mortality is restricted to patients with moderate organ failure. *Ann Intensive Care*
**2017**, *7*, 44.
6. Bermejo-Martín, J.F.; Tamayo, E.; Ruiz, G.; Andaluz-Ojeda, D.; Herrán-Monge, R.; Muriel-Bombín, A.; Fe Muñoz, M.; Heredia-Rodríguez, M.; Citores, R.; Gómez-Herreras, J.; et al. Circulating neutrophil counts and mortality in septic shock. *Crit Care*
**2014**, *18*, 407.
7. Bermejo-Martin, J.F.; Martin-Loeches, I.; Rello, J.; Antón, A.; Almansa, R.; Xu, L.; Lopez-Campos, G.; Pumarola, T.; Ran, L.; Ramirez, P.; et al. Host adaptive immunity deficiency in severe pandemic influenza. *Crit Care*
**2010**, *14*, R167.
8. Davenport, E.E.; Burnham, K.L.; Radhakrishnan, J.; Humburg, P.; Hutton, P.; Mills, T.C.; Rautanen, A.; Gordon, A.C.; Garrard, C.; Hill, A.V.S.; et al. Genomic landscape of the individual host response and outcomes in sepsis: a prospective cohort study. *Lancet Respir Med*
**2016**, *4*, 259–271.
9. Bermejo-Martin, J.F.; Andaluz-Ojeda, D.; Martin-Fernandez, M.; Aldecoa, C.; Almansa, R. Composed endotypes to guide antibiotic discontinuation in sepsis. *Crit Care*
**2019**, *23*, 140.
10. Menéndez, R.; Méndez, R.; Almansa, R.; Ortega, A.; Alonso, R.; Suescun, M.; Ferrando, A.; Feced, L.; Bermejo-Martin, J.F. Simultaneous Depression of Immunological Synapse and Endothelial Injury is Associated with Organ Dysfunction in Community-Acquired Pneumonia. *J Clin Med*
**2019**, *8*.

#### **2.4.2. Cell Therapies in Sepsis: New Insights** 

H.GonzalezCMastersonJ.G.Laffey

##### **Background** 

Acute respiratory distress syndrome (ARDS) is a life-threating critical illness, characterised by a severe pro-inflammatory response causing lung injury, which can progress to multiple organ failure and death (1). ARDS is seen in 23% of mechanically ventilated patients in the ICU (2), and represents 10.4% of global ICU admissions, with small variances between continents (3). Despite the improvement in critical care support in the last number of years, 40% of ARDS patients die in hospital. The need for an effective and safe treatment for ARDS promotes the search of alternative approaches. Here we focus on cell therapy, specifically mesenchymal stem cells, which have demonstrated potential for ARDS.

##### **Pre-Clinical Studies Supporting Cell Therapy** 

MSCs are adult derived stem cells that can be isolated from various sources as bone marrow (BM), adipose tissue (AD) or umbilical cord (UC). MSCs present low levels of MHC I expression and virtually no MHC II or costimulatory molecules, allowing allogenic transplant without immune rejection (4). MSCs have potent immunomodulatory effects; reprogramming the immune response to reduce tissue damage and enhance their tissue repair capacity. From a logistic point of view, the multiple sources and their capacity of stable in vitro expansion, whilst maintaining an undifferentiated state and retaining activity, permits the escalated production of these cells for clinical use. 

In preclinical ARDS models, MSCs improve survival in LPS-induced acute lung injury, decreasing oxidative stress and inflammation (5). Devaney et al. demonstrated that MSCs reduce lung bacterial burden and improve survival in an *E. coli* pneumonia model (6) and Mei et al. reported similar effects in a systemic infection model (7). MSC’s capacity to improve damage caused by mechanical ventilation has been reported as well, whereby Curley et al. demonstrated enhanced recovery after MSC administration in a ventilator-induced lung injury model (8).

##### **Clinical Studies** 

In recent years, phase 1 clinical trials have reported on the safety of MSCs for different diseases (9). Wilson et al. have shown in ‘The STem cells for ARDS Treatment’ phase I pilot trial (START; NCT01775774), that intravenous administration of bone-marrow derived MSCs in nine patients with moderate to severe ARDS did not present any safety issues (10). The same group in early 2019 presented the results from a phase 2a safety trial (NCT02097641) where 60 patients received a single dose of 10 million BM-MSCs/kg or placebo. While there were no adverse effects reported related to the MSCs administration, mortality was non-significantly higher in the treatment group (11). Recently, positive safety outcomes from the phase 1/2 MultiStem^®^ Therapy in Acute Respiratory Distress Syndrome trial (MUST-ARDS; NCT02611609) were announced with an inference toward efficacy of high doses of MSCs in ARDS patients [Bellingan, 2019]. Ongoing clinical studies include the ‘Repair of Acute Respiratory Distress Syndrome by Stromal Cell Administration’ (REALIST; NCT03042143), where patients are receiving human umbilical cord derived MSCs. MultiStem^®^ therapy is also being investigated in a phase 1 trial in patients with pneumonia-induced ARDS (ONE-BRIDGE; NCT03807804). In late 2019 Japan’s Ministry of Health, Labour and Welfare conferred orphan regenerative medicine designation to MultiStem^®^ therapy for ARDS. 

Clinical trials in syndromes related to ARDS such as sepsis and septic shock have been performed. A Russian clinical trial using mesenchymal cells in septic shock and severe neutropenia (RUMCESS, NCT01849237) suggested an improvement in the short-term survival in septic shock patients after MSC administration (12). Recently, the ‘Cellular immunotherapy for septic shock’ (CISS; NCT02421484) phase 1 trial results has demonstrated the safety and tolerability of BM-MSCs up to 250 × 10^6^ cells in septic shock patients (13). The same research team is developing a phase 2 clinical trial (CISS2; NCT03369275) enrolling 114 participants to confirm the previous results. There are more clinical trials ongoing at the moment, targeting the treatment of severe infections with MSCs (CHOCMS; NCT02883803). 

##### **Challenges and Future Opportunities** 

Our understanding of the biology of MSCs and their mechanisms of action in ARDS has vastly improved in the last number of years, but many questions still remain unanswered. The translation of this therapy to the clinic still presents some issues that have to be addressed to maximize therapeutic potential. Differences between batches of cells, optimizing cell cryopreservation and thawing approaches, all require further research. The administration time of the MSCs appears to be crucial for the outcome, and most of the pre-clinical studies have focused on the early administration of the cells, a situation not always possible in the clinic. 

Despite the remaining challenges for the future, new studies are exploring different approaches to solve the current limitations and improve MSC effcacy. Recently Jerkic et al. showed improved efficacy using UC-MSCs over-expressing IL-10 in resolving *E. coli* lung infection in a pneumosepsis rodent model (14). There is also ongoing research involving the use of MSC products instead of the cell itself, avoiding any perceived problems with administration of cells, such as pulmonary circulation micro emboli or infusion toxicity. Studies have demonstrated the therapeutic potential of the whole MSC secretome, while others have shown the potential for purified MSC-derived extracellular vesicles and exosomes in lung injury models (15, 16). Also, alternative delivery routes are under study to facilitate their use in the clinic. Recently McCarthy et al. has shown that nebulised MSC conditioned media retains antibacterial properties against clinically relevant pathogens (17).

##### **Conclusions** 

MSCs demonstrate considerable therapeutic promise for ARDS. Increasing insights exist into their diverse mechanisms of action, including their modulation of the immune response, ability to reduce host injury by attenuating the pro-inflammatory response, increasing bacterial clearance, and facilitating repair. Encouraging safety data is emerging from phase I and phase II clinical trials. However, better characterisation of MSCs will address current knowledge gaps, and should maximize the therapeutic potential of MSCs for ARDS.

###### **References** 

1. Ware, L.B.; Matthay, M.A. The Acute Respiratory Distress Syndrome. *N. Engl. J. Med.*
**2000**, *342*, 1334–1349.
2. McNicholas, B.A.; Rooney, G.M.; Laffey, J.G. Lessons to learn from epidemiologic studies in ARDS. *Curr. Opin. Crit. Care*
**2018**, *24*, 41–48.
3. Bellani, G.; Laffey, J.G.; Pham, T.; Fan, E.; Brochard, L.; Esteban, A.; Gattinoni, L.; Van Haren, F.; Larsson, A.; McAuley, D.F.; et al. Epidemiology, Patterns of Care, and Mortality for Patients With Acute Respiratory Distress Syndrome in Intensive Care Units in 50 Countries. *JAMA*
**2016**, *315*, 788–800.
4. Nasef, A.; Mathieu, N.; Chapel, A.; Frick, J.; Fran??ois, S.; Mazurier, C.; Boutarfa, A.; Bouchet, S.; Gorin, N.-C.; Thierry, D.; et al. Immunosuppressive Effects of Mesenchymal Stem Cells: Involvement of HLA-G. *Transplant.*
**2007**, *84*, 231–237.
5. Pedrazza, L.; Cunha, A.A.; Luft, C.; Nunes, N.K.; Schimitz, F.; Gassen, R.B.; Breda, R.V.; Donadio, M.V.F.; Wyse, A.T.D.S.; Pitrez, P.M.C.; et al. Mesenchymal stem cells improves survival in LPS-induced acute lung injury acting through inhibition of NETs formation. *J. Cell. Physiol.*
**2017**, *232*, 3552–3564.
6. Devaney, J.; Horie, S.; Masterson, C.; Elliman, S.; Barry, F.; O’Brien, T.; Curley, G.; O’Toole, D.; Laffey, J.G. Human mesenchymal stromal cells decrease the severity of acute lung injury induced by E. coli in the rat. *Thorax*
**2015**, *70*, 625–635.
7. Mei, S.H.J.; Haitsma, J.J.; Dos Santos, C.C.; Deng, Y.; Lai, P.F.H.; Slutsky, A.S.; Liles, W.C.; Stewart, D.J. Mesenchymal Stem Cells Reduce Inflammation while Enhancing Bacterial Clearance and Improving Survival in Sepsis. *Am. J. Respir. Crit. Care Med.*
**2010**, *182*, 1047–1057.
8. Curley, G.F.; Ansari, B.; Hayes, M.; Devaney, J.; Masterson, C.; Ryan, A.; Barry, F.; O’Brien, T.; Toole, D.O.; Laffey, J.G. Effects of Intratracheal Mesenchymal Stromal Cell Therapy during Recovery and Resolution after Ventilator-induced Lung Injury. *Anesthesiol.*
**2013**, *118*, 924–932.
9. Walter, J.; Ware, L.B.; A Matthay, M. Mesenchymal stem cells: mechanisms of potential therapeutic benefit in ARDS and sepsis. *Lancet Respir. Med.*
**2014**, *2*, 1016–1026.
10. Wilson, J.G.; Liu, K.D.; Zhuo, H.; Caballero, L.; McMillan, M.; Fang, X.; Cosgrove, K.; Vojnik, R.; Calfee, C.S.; Lee, J.W.; et al. Mesenchymal stem (stromal) cells for treatment of ARDS: a phase 1 clinical trial. *Lancet Respir. Med*. **2015**, *3*, 24–32.
11. Hibbert, K.A.; Ma, M.; Cs, C.; H, Z.; Bt, T.; Jg, W.; Je, L.; Aj, R.; Je, G.; Jp, W.-K.; et al. F1000Prime recommendation of Treatment with allogeneic mesenchymal stromal cells for moderate to severe acute respiratory distress syndrome (START study): a randomised phase 2a safety trial. *F1000—Post-publication peer review of the biomedical literature*
**2019**, *7*.
12. Galstian, G.M.; Parovichnikova, E.N.; Makarova, P.M.; Kuzmina, L.A.; Troitskaya, V.V.; Gemdzhian, E.; Drize, N.I.; Savchenko, V.G. The Results of the Russian Clinical Trial of Mesenchymal Stromal Cells (MSCs) in Severe Neutropenic Patients (pts) with Septic Shock (SS) (RUMCESS trial). *Blood*
**2015**, *126*, 2220.
13. A McIntyre, L.; Watpool, I.; Schlosser, K.; Stewart, D.J.; Mei, S.H.J.; Courtman, D.; Granton, J.; Marshall, J.; Dos Santos, C.; Walley, K.R.; et al. Cellular Immunotherapy for Septic Shock. A Phase I Clinical Trial. *Am. J. Respir. Crit. Care Med.*
**2018**, *197*, 337–347.
14. Jerkic, M.; Masterson, C.; Ormesher, L.; Gagnon, S.; Goyal, S.; Rabani, R.; Otulakowski, G.; Zhang, H.; Kavanagh, B.P.; Laffey, J.G. Overexpression of IL-10 Enhances the Efficacy of Human Umbilical-Cord-Derived Mesenchymal Stromal Cells in E. coli Pneumosepsis. *J. Clin. Med.*
**2019**, *8*, 847.
15. Ionescu, L.; Byrne, R.N.; Van Haaften, T.; Vadivel, A.; Alphonse, R.S.; Rey-Parra, G.J.; Weissmann, G.; Hall, A.; Eaton, F.; Thébaud, B. Stem cell conditioned medium improves acute lung injury in mice: in vivo evidence for stem cell paracrine action. *Am. J. Physiol. Cell. Mol. Physiol.*
**2012**, *303*, L967–L977.
16. Zhu, Y.-G.; Feng, X.-M.; Abbott, J.; Fang, X.-H.; Hao, Q.; Monsel, A.; Qu, J.-M.; Matthay, M.A.; Lee, J.W. Human mesenchymal stem cell microvesicles for treatment of Escherichia coli endotoxin-induced acute lung injury in mice. *STEM CELLS*
**2014**, *32*, 116–125.
17. McCarthy, S.D.; Horgan, E.; Ali, A.; Masterson, C.; Laffey, J.G.; MacLoughlin, R.; O’Toole, D. Nebulized Mesenchymal Stem Cell Derived Conditioned Medium Retains Antibacterial Properties Against Clinical Pathogen Isolates. *J. Aerosol Med. Pulm. Drug Deliv.*
**2019**.

### 2.5. SESION V: Severe Pulmonary Infections

#### **2.5.1. Animal Models in VAP** 

GaleraAna MotosHospital Clinic de Barcelona, Barcelona, Spain

Ventilator-associated pneumonia (VAP) is a type of lower respiratory tract infection that develops ≥48 h following mechanical ventilation (1,2). VAP is the leading cause of mortality and morbidity in critically ill patients among nosocomial infections (1, 2). VAP may be caused by a variety of pathogens and, in many patients, more than one pathogen can be isolated (3,4). The most frequent causative pathogens are Gram-negative bacilli, such as *Pseudomonas aeruginosa*, *Escherichia coli, Klebsiella pneumoniae,* or *Acinetobacter baumannii*, while *Staphylococcus aureus* is the most common Gram-positive pathogen (5). Bacterial resistance to multiple antibiotics has become increasingly frequent in patients with VAP by Gram-negative pathogens and existing antibiotic therapies are often ineffective. Patients at risk of being colonized by multi-drug resistant pathogens are extremely heterogeneous, often present several co-morbidities, and many receive antibiotics prior to and during the course of their hospitalization (6). Additionally, the pattern of resistance varies widely among institutions and departments (7). As a consequence, the mortality associated with VAP has not substantially decreased over the last decades, despite improvements in treatment and intensive care support.

In the last 20 years, different animal models have allowed us to elucidate the pathogenesis of VAP, but also to test novel therapies and equipment as well as study the pharmacology (8). Hence, animal models have become a decisive step between in vitro testing and clinical studies. In this scenario, a wide variety of animal models of pneumonia has been developed (8,9). Most of the experimental studies are performed in mammals; specifically, in the last decades, studies in rodents have gained increasing interest. These small animal models are commonly used because their size, rapid reproductive rate, potential for extensive genome manipulation and limited costs (10). Nevertheless, significant anatomical and physiological differences between rodents and humans have been noted (11). In addition, long term mechanical ventilation and ICU-related concomitant conditions are challenging. Also, the severity of the infection can quickly kill small animals, which hinders the long-term effectiveness of novel therapies (10,12). 

Therefore, several models in large animals such dogs, cats, pigs, sheep or non-human primates have been described (13–17). Currently, dogs, cats and non-human primates have been replaced to sheep and pigs due to the ethical requirements related to the care and use of research animals and the public concern for these pet species. Large animal models are close to human anatomical features admit prolonged mechanical ventilation and reproduce intensive care settings (12). However, sample size of these studies is limited by their significant expenses, intensive labour and high time-consuming. Furthermore, large animal models are young healthy animals without comorbidities and deeply sedated throughout the experimental studies; a different scenario that we find in the intensive care units. Contrarily, these circumstances help to test different hypotheses and novel approaches without prior medication interference or comorbidities (12). 

In this context, animal models constitute the basis for the successful development of novel therapies and innovative preventive and therapeutic approaches for VAP, although differences with respect to the VAP in humans must be taken into account before drawing translational conclusions (18,19).

##### **References** 

1. Torres, A.; Niederman, M.S.; Chastre, J.; Ewig, S.; Fernandez-Vandellos, P.; Hanberger, H.; Kollef, M.; Li Bassi, G.; Luna, C.M.; Martin-Loeches, I.; et al. International ERS/ESICM/ESCMID/ALAT guidelines for the management of hospital-acquired pneumonia and ventilator-associated pneumonia: Guidelines for the management of hospital-acquired pneumonia (HAP)/ventilator-associated pneumonia (VAP) of the European Respiratory Society (ERS), European Society of Intensive Care Medicine (ESICM), European Society of Clinical Microbiology and Infectious Diseases (ESCMID) and Asociacion Latinoamericana del Torax (ALAT). *Eur. Respir. J*. **2017**, *50*, pii1700582.
2. Kalil, A.C.; Metersky, M.L.; Klompas, M.; Muscedere, J.; Sweeney, D.A.; Palmer, L.B.; Napolitano, L.M.; O’Grady, N.P.; Bartlett, J.G.; Carratalà, J.; et al. Management of Adults With Hospital-acquired and Ventilator-associated Pneumonia: 2016 Clinical Practice Guidelines by the Infectious Diseases Society of America and the American Thoracic Society. *Clin. Infect. Dis*. **2016**, *63*, e61–e111.
3. Warren, D.K.; Shukla, S.J.; Olsen, M.A.; Kollef, M.H.; Hollenbeak, C.S.; Cox, M.J.; Cohen, M.M.; Fraser, V.J. Outcome and attributable cost of ventilator-associated pneumonia among intensive care unit patients in a suburban medical center*. *Crit. Care Med.*
**2003**, *31*, 1312–1317.
4. Rello, J.; Ollendorf, D.A.; Oster, G.; Vera-Llonch, M.; Bellm, L.; Redman, R.; Kollef, M.H. Epidemiology and outcomes of ventilator-associated pneumonia in a large US database. *Chest*
**2002**, *122*, 2115–2121.
5. Vincent, J.-L.; Rello, J.; Marshall, J.; Silva, E.; Anzueto, A.; Martin, C.D.; Moreno, R.; Lipman, J.; Gomersall, C.; Sakr, Y.; et al. International Study of the Prevalence and Outcomes of Infection in Intensive Care Units. *JAMA*
**2009**, *302*, 2323.
6. O Depuydt, P.; Vandijck, D.M.; A Bekaert, M.; Decruyenaere, J.M.; I Blot, S.; Vogelaers, D.P.; Benoit, D.D. Determinants and impact of multidrug antibiotic resistance in pathogens causing ventilator-associated-pneumonia. *Crit. Care*
**2008**, *12*, R142.
7. Rello, J.; Sa-Borges, M.; Correa, H.; Leal, S.R.; Baraibar, J. Variations in etiology of ventilator-associated pneumonia across four treatment sites: implications for antimicrobial prescribing practices*. Am. J. Respir. Crit. Care Med*. **1999**, *160*, 608–613.
8. Luna, C.M.; Sibila, O.; Agustí, C.; Torres, A. Animal models of ventilator-associated pneumonia. *Eur. Respir. J.*
**2009**, *33*, 182–188.
9. Pulido, L.; Burgos, D.; Morato, J.G.; Luna, C.M. Does animal model on ventilator-associated pneumonia reflect physiopathology of sepsis mechanisms in humans? *Ann. Transl. Med.*
**2017**, *5*, 452.
10. Mizgerd, J.P.; Skerrett, S.J. Animal models of human pneumonia. *Am. J. Physiol. Cell. Mol. Physiol.*
**2008**, *294*, L387–L398.
11. Kling, M.A. A Review of Respiratory System Anatomy, Physiology, and Disease in the Mouse, Rat, Hamster, and Gerbil. *Veter- Clin. North Am. Exot. Anim. Pr.*
**2011**, *14*, 287–337.
12. Bielen, K.; Jongers, B. ’S; Malhotra-Kumar, S.; Jorens, P.G.; Goossens, H.; Kumar-Singh, S. Animal models of hospital-acquired pneumonia: current practices and future perspectives. *Ann. Transl. Med.*
**2017**, *5*, 132.
13. Marquette, C.-H.; Wermert, D.; Wallet, F.; Copin, M.-C.; Tonnel, A.-B. Characterization of an animal model of ventilator-acquired pneumonia. *Chest*
**1999**, *115*, 200–209.
14. Higuchi, J.H.; Coalson, J.J.; Johanson, W.G. Bacteriologic diagnosis of nosocomial pneumonia in primates. Usefulness of the protected specimen brush. *Am. Rev. Respir. Dis.* 1982, *125*.
15. Martínez-Olondris, P.; Sibila, O.; Agustí, C.; Rigol, M.; Soy, D.; Esquinas, C.; Piner, R.; Luque, N.; Guerrero, L.; Quera, M.A.; et al. An experimental model of pneumonia induced by methicillin-resistant Staphylococcus aureus in ventilated piglets. *Eur. Respir. J.*
**2010**, *36*, 901–906.
16. Bassi, G.L.; Rigol, M.; Marti, J.-D.; Saucedo, L.; Ranzani, O.T.; Roca, I.; Cabañas, M.; Muñoz, L.; Giunta, V.; Luque, N.; et al. A Novel Porcine Model of Ventilator-associated Pneumonia Caused by Oropharyngeal Challenge with Pseudomonas aeruginosa. *Anesthesiol.*
**2014**, *120*, 1205–1215.
17. Shure, D.; Moser, K.M.; Konopka, R. Transbronchial needle aspiration in the diagnosis of pneumonia in a canine model. *Am. Rev. Respir. Dis.* 1985, *131*.
18. Metersky, M.; Waterer, G. Can animal models really teach us anything about pneumonia? Con. *Eur. Respir. J.*
**2020**, *55*, 1901525.
19. Orihuela, C.J.; Maus, U.A.; Brown, J.S. Can animal models really teach us anything about pneumonia? Pro. *Eur. Respir. J.*
**2020**, *55*, 1901539.

#### **2.5.2. Hospital-Acquired Pneumonia in Non-Ventilated Patients** 

FerrerMiquelRIICU, Department of Pneumology, Respiratory Institute, Hospital Clínic of Barcelona, IDIBAPS, CibeRes, Universitat de Barcelona

Most studies on hospital acquired pneumonia (HAP) are focused in mechanically-ventilated patients. The incidence of pneumonia in this population, namely ventilator-associated pneumonia (VAP), is higher compared with non-ventilated patients. VAP has elevated morbidity and mortality, and the responsible micro-organisms are well defined in VAP. Therefore, it is likely that there may be bias due to extrapolation this information to non-ventilated HAP.

Few studies evaluated HAP in non-ventilated patients, mainly outside the intensive care unit (ICU) until recently. The rate of etiologic diagnosis of pneumonia is higher in VAP. A multicentre Spanish study reported higher proportions of “community” and environmental organisms, and lower proportions of non-fermenting (NF) gram-negative bacilli (NF-GNB), compared with series on VAP. However, multi-drug resistant (MDR) pathogens are sufficiently frequent in non-ventilated patients to be considered in the empirical antibiotic treatment. In addition, the outcomes of HAP in non-ventilated patients of these series are better compared with VAP patients.

Non-ventilated patients outside the ICU are expected to have less severe underlying clinical condition than ICU patients. Conversely, ICU patients are expected to have more frequent risk factors for specific pathogens. Therefore, several studies addressed the question on whether the different aetiology and worse outcome of VAP depend on previous intubation or higher severity of the host. 

It is estimated that only 30% patients admitted in ICUs are mechanically-ventilated for more than 24 h. Among published series on ICU-acquired pneumonia, the proportion of non ventilator-associated HAP ranges between 4% and 73%. In general, patients with non-ventilator ICU-acquired pneumonia have: (1) similar baseline severity than VAP patients, although less organ system failure; (2) older age, possibly because the oldest patients are more likely to have previous do-not-intubate decisions; and (3) pneumonia occurred earlier in the ICU than VAP, because they may have less protection from aspiration while they are critically ill and because endotracheal intubation may facilitate removal of bronchial secretions and delay infection.

As in HAP outside the ICU, there is a lower rate of etiologic diagnosis patients with non-ventilator ICU-acquired pneumonia than in VAP patients, probably due to less availability of LRT samples of high quality for microbiology, and less bacterial burden due to shorter previous critical illness. The most frequent bacterial isolated are enteric GNB, NF-GNB and *Staphylococcus aureus*. Although these microbes are more frequency isolated in VAP patients, there are no major differences in the types of isolates when these rates are corrected for patients with aetiology. Community-acquired pathogens such as *Streptococcus pneumoniae* appear to be more frequent in NV-ICUAP. Therefore, aetiology may depend on both patients’ underlying severity and previous intubation. Therefore, similar empiric antibiotic treatment of pneumonia is advisable between ventilated and non-ventilated ICU patients. 

Finally, the long-term mortality is slightly lower in patients with non-ventilator ICU-acquired pneumonia; however, when adjusting for confounders, the mortality is similar in both types of patients, probably because of the similar baseline severity and the limited attributable mortality of VAP. However, patients with non-ventilator ICU-acquired pneumonia who need subsequent intubation have a particularly higher mortality; they represent a group of patients with non response to the empiric treatment.

##### **References** 

1. Torres, A.; Niederman, M.S.; Chastre, J.; Ewig, S.; Fernandez-Vandellos, P.; Hanberger, H.; Kollef, M.; Li Bassi, G.; Luna, C.M.; Martin-Loeches, I.; et al. International ERS/ESICM/ESCMID/ALAT guidelines for the management of hospital-acquired pneumonia and ventilator-associated pneumonia: Guidelines for the management of hospital-acquired pneumonia (HAP)/ventilator-associated pneumonia (VAP) of the European Respiratory Society (ERS), European Society of Intensive Care Medicine (ESICM), European Society of Clinical Microbiology and Infectious Diseases (ESCMID) and Asociacion Latinoamericana del Torax (ALAT). *Eur. Respir. J*. **2017**, *50*, 1700582.
2. Kalil, A.C.; Metersky, M.L.; Klompas, M.; Muscedere, J.; Sweeney, D.A.; Palmer, L.B.; Napolitano, L.M.; O’Grady, N.P.; Bartlett, J.G.; Carratalà, J.; et al. Management of Adults With Hospital-acquired and Ventilator-associated Pneumonia: 2016 Clinical Practice Guidelines by the Infectious Diseases Society of America and the American Thoracic Society. *Clin. Infect. Dis*. **2016**, 63, e61–e111.
3. Esperatti, M.; Ferrer, M.; Theessen, A.; Liapikou, A.; Valencia, M.; Saucedo, L.M.; Zavala, E.; Welte, T.; Torres, A. Nosocomial Pneumonia in the Intensive Care Unit Acquired during Mechanical Ventilation or Not. *Am. J. Respir. Crit. Care Med*. **2010**, *182*, 1533–1539.
4. Kohlenberg, A.; Schwab, F.; Behnke, M.; Geffers, C.; Gastmeier, P. Pneumonia associated with invasive and noninvasive ventilation: an analysis of the German nosocomial infection surveillance system database. *Intensive Care Med*. **2010**, *36*, 971–978.
5. Sopena, N.; Sabria, M.; Neunos 2000 Study Group. Multicenter study of hospital-acquired pneumonia in non-ICU patients. *Chest*. **2005**, *127*, 213–219.
6. Weber, D.J.; Rutala, W.A.; Sickbert-Bennett, E.E.; Samsa, G.P.; Brown, V.; Niederman, M.S. Microbiology of ventilator-associated pneumonia compared with that of hospital-acquired pneumonia. *Infect Control Hosp. Epidemiol*. **2007**, *28*, 825–831.
7. Karhu, J.; la-Kokko, T.I.; Ylipalosaari, P.; Ohtonen, P.; Laurila, J.J.; Syrjala, H. Hospital and long-term outcomes of ICU-treated severe community- and hospital-acquired, and ventilator-associated pneumonia patients. *Acta Anaesthesiol. Scand*. **2011**, *55*, 1254–1260.
8. Talbot, G.H.; Das, A.; Cush, S.; Dane, A.; Wible, M.; Echols, R.; Torres, A.; Cammarata, S.; Rex, J.H.; Powers, J.H.; et al. Evidence-Based Study Design for Hospital-Acquired Bacterial Pneumonia and Ventilator-Associated Bacterial Pneumonia. *J. Infect Dis*. **2019**, *219*, 1536–1544.

#### **2.5.3. Hyperoxemia as a Risk Factor for Ventilator-Associated Pneumonia** 

NseirSaad^1^,^2^1Lille University Hospital, Critical Care Center, F-59000 Lille, France; s-nseir@chru-lille.fr2Lille University, U995-LIRIC-Lille Inflammation Research International Center, F-59000 Lille, France

High concentrations of oxygen are routinely used during the supportive care in critically ill patients. Although existing data remain conflicting regarding the risk related to hyperoxemia in critical care, results from the latest clinical studies suggest that hyperoxemia is probably associated with worse outcomes in some critically ill patients. Potential reasons for these conflicting results are significant heterogeneity between the studies regarding hyperoxemia definition, time of assessment, timing and duration of hyperoxemia. Two meta-analyses [1, 2] suggest that hyperoxemia is associated with increased mortality in different populations of critically ill patients (8,9), including post-cardiac arrest [OR =1.42 (1.04–1.92), I2 = 68%], stroke [OR =1.23 (1.06–1.43), I2 = 0%], and traumatic brain injury [OR =1.41 (1.03–1.94), I2 = 65%]. In addition, a recent single-center randomized controlled trial reported increased mortality in patients with conventional therapy, as compared with conservative oxygen therapy [3].

Recent studies suggest a relationship between hyperoxemia and ventilator-associated pneumonia (VAP). Hyperoxemia is responsible for denitrogenation phenomena, and inhibition of surfactant production, promoting atelectasis in mechanically ventilated patients. Further, hyperoxemia impairs the efficacy of alveolar macrophages to migrate, phagocyte and kill bacteria. Oxygen can also cause pulmonary-specific toxic effect called hyperoxic acute lung injury leading to longer duration of mechanical ventilation [4]. All these hyperoxic effects are well-known risk factors for VAP. Entezari and colleagues [5] showed that prolonged exposure to hyperoxaemia impaired macrophage capacities to phagocytose *Pseudomonas aeruginosa*. Another study found hyperoxaemia to be associated with increased serum concentration of high mobility group box 1 (HMGB-1), and increased mortality in mice infected with *P aeruginosa*. In a mice model exposed to hyperoxaemia, ascorbic acid supplementation significantly improved bacterial clearance of *P aeruginosa*, and reduced accumulation of HMGB-1, and reactive oxygen species in the lung. Our group performed a retrospective analysis of prospectively collected data in a cohort of 503 patients receiving invasive mechanical ventilation for more than 48 h [6]. Hyperoxaemia was identified as an independent risk factor for ventilator-associated pneumonia (OR 1.1, 95% CI 1.04–1.2 per day, p=0.004). However, two recent randomized controlled trials evaluated the impact of conservative oxygen strategy versus a liberal strategy, but did not confirm the role of hyperoxemia in lower respiratory tract infection occurrence. 

Further large prospective studies in carefully selected groups of patients are required to confirm the potential role of hyperoxemia in VAP pathogenesis and to evaluate the impact of a conservative oxygen strategy vs. a conventional strategy on the incidence of VAP.

##### **References** 

1. Damiani, E.; Adrario, E.; Girardis, M.; Romano, R.; Pelaia, P.; Singer, M.; Donati, A. Arterial hyperoxia and mortality in critically ill patients: a systematic review and meta-analysis. *Crit. Care*
**2014**, *18*, 711.
2. Helmerhorst, H.J.F.; Schultz, M.J.; van der Voort, P.H.J.; Bosman, R.J.; Juffermans, N.P.; de Wilde, R.B.P.; van den Akker-van Marle, M.E.; van Bodegom-Vos, L.; de Vries, M.; Eslame, S.; et al. Effectiveness and Clinical Outcomes of a Two-Step Implementation of Conservative Oxygenation Targets in Critically Ill Patients: A Before and After Trial. *Crit. Care Med*. **2015**, *44*, 554–563.
3. Girardis, M.; Busani, S.; Damiani, E.; Donati, A.; Rinaldi, L.; Marudi, A.; Morelli, A.; Antonelli, M.; Singer, M. Faculty of 1000 evaluation for Effect of Conservative vs. Conventional Oxygen Therapy on Mortality Among Patients in an Intensive Care Unit: The Oxygen-ICU Randomized Clinical Trial. *F1000—Post-Publ. Peer Rev. Biomed. Lit.*
**2016**, *316*, 1583–1589.
4. Jaffal, K.; Six, S.; Zerimech, F.; Nseir, S. Relationship between hyperoxemia and ventilator associated pneumonia. *Ann. Transl. Med.*
**2017**, *5*, 453.
5. Entezari, M.; Javdan, M.; Antoine, D.J.; Morrow, D.M.; Sitapara, R.A.; Patel, V.; Wang, M.; Sharma, L.; Gorasiya, S.; Zur, M.; et al. Inhibition of extracellular HMGB1 attenuates hyperoxia-induced inflammatory acute lung injury. *Redox Boil.*
**2014**, *2*, 314–322.
6. Six, S.; Jaffal, K.; LeDoux, G.; Jaillette, E.; Wallet, F.; Nseir, S. Hyperoxemia as a risk factor for ventilator-associated pneumonia. *Crit. Care*
**2016**, *20*, 195.

#### **2.5.4. Role of Endotracheal Tube Biofilm in VAP** 

BaratLaia FernándezResearch laboratory coordinator, Ciberes-IDIBAPS, University of Barcelona

Among 65–80% of human infections are associated to biofilms, especially in respiratory infections or those associated with indwelling medical devices such as endotracheal tubes (ETT) [1,2]. Biofilms develop rapidly following endotracheal intubation, with well-organized antibiotic-tolerant microbial structures that represent a persistent source of pathogens in the critically ill patient [3]. Whether this colonization of the ETT is a direct cause of ventilator associated pneumonia (VAP) or a concomitant fact associated with the spread of microorganisms from the tracheobronchial tree to the tube during intubation is difficult to assess because ETTs can only be obtained after extubation, since re-intubation itself represents an independent risk factor for nosocomial pneumonia [4]. However, ETT-biofilm is considered one of the multiple pathophysiological mechanisms that lead to (VAP) and/or its relapses [5].

Animal and clinical trials have already tested biofilm-preventive measures. In particular, the Silver-coated ETTs and the Mucus Shaver evidenced successful results in terms of biofilm prevention [6–8]. Nevertheless, these preventive measures are associated with the increased costs, not easy to implement and thus they are not yet universally adopted. 

Recently, studies have evidenced that VAP patients have lower biodiversity in lung microbiota in comparison to the same subjects at intubation [9]. This finding sheds light into the potential role of lung and ETT microbiota in VAP suggesting that VAP classical pathophysiology should be re-evaluated. New parameters should be incoporated into the VAP equation such as: the imbalance in the lung microbiome caused by an ETT and how this imbalance is propagated within ETT-biofilm also influenced by antimicrobials, usually present in the context of ICU patients at risk of respiratory infections.

Mature biofilms exhibit antimicrobial tolerance and harbor resistant bacteria but there is scarce evidence on the effect of systemic, aerosolized or combined regimens of antimicrobials on ETT-biofilm formation during mechanical ventilation. Along the last 12 years, in our research laboratory we have studied preventive and treatment strategies for ETT-biofilms in mechanically ventilated subjects with pneumonia due to MRSA, *Pseudomonas aeruginosa* or *Streptococcus pneumoniae*. As a result, and through the standardizing of the methodology we have unveiled novel aspects of in vivo ETT-biofilms development [10], as well as the effect of different preventive [11,12] and treatment strategies some of them already applied in clinical practice^13^, or close to, whilst others in preclinical phases.

##### **References** 

1. Lebeaux, D.; Chauhan, A.; Rendueles, O.; Beloin, C. From in vitro to in vivo Models of Bacterial Biofilm-Related Infections. *Pathogens*
**2013**, *2*, 288–356.
2. Høiby, N.; Bjarnsholt, T.; Moser, C.; Bassi, G.L.; Coenye, T.; Donelli, G.; Hall-Stoodley, L.; Holá, V.; Imbert, C.; et al. ESCMID guideline for the diagnosis and treatment of biofilm infections 2014. *Clin. Microbiol. Infect.*
**2015**, *21* (Suppl. 1), S1–25.
3. Costerton, J.W.; Stewart, P.S.; Greenberg, E.P. Bacterial biofilms: a common cause of persistent infections. *Science*
**1999**, *21*, 1318–1322.
4. Torres, A.; Gatell, J.M.; Aznar, E.; el-Ebiary, M.; Puig de la Bellacasa, J.; González, J.; Ferrer, M.; Rodriguez-Roisin, R. Re-intubation increases the risk of nosocomial pneumonia in patients needing mechanical ventilation. *Am. J. Respir. Crit. Care Med.*
**1995**, *152*, 137–141.
5. Fernandez-Barat, L.; Torres, A. Biofilms in ventilator-associated pneumonia. *Future Microbiol.*
**2016**, *11*, 1599–1610.
6. Kollef, M.H.; Afessa, B.; Anzueto, A.; Veremakis, C.; Kerr, K.M.; Margolis, B.D.; Craven, D.E.; Roberts P.R.; Arroliga, A.C.; Hubmayr, R.D.; et al. Silver-coated endotracheal tubes and incidence of ventilator-associated pneumonia: the NASCENT randomized trial. *JAMA*
**2008**, *300*, 805–813.
7. Berra, L.; Coppadoro, A.; Bittner, E.A.; Kolobow, T.; Laguerriere, P.; Pohlmann, J.R.; Bramati, S.; Moss, J.; Presenti, A. A clinical assessment of the Mucus Shaver: a device to keep the endotracheal tube free from secretions. *Crit. Care Med.*
**2012**, *40*, 119–124.
8. Tokmaji, G.; Vermeulen, H.; Müller, M.C.; Kwakman, P.H.; Schultz, M.J.; Zaat, S.A. Silver-coated endotracheal tubes for prevention of ventilator-associated pneumonia in critically ill patients. *Cochrane Database Syst. Rev.*
**2015**, *8*, CD009201.
9. Zakharkina, T.; Martin-Loeches, I.; Matamoros, S.; Povoa, P.; Torres, A.; Kastelijn, J.B.; Hofstra, J.J.; de Wever, B.; de Jong, M.; Schultz, M.J.; et al. The dynamics of the pulmonary microbiome during mechanical ventilation in the intensive care unit and the association with occurrence of pneumonia. *Thorax.*
**2017**, *72*, 803–810.
10. Fernandez-Barat, L.; Li, B.G.; Ferrer, M.; Bosch, A.; Calvo, M.; Vila, J.; Gabarrús, A.; Martínez-Olondris, P.; Rigol, M.; Esperatti, M.; et al. Direct analysis of bacterial viability in endotracheal tube biofilm from a pig model of methicillin-resistant Staphylococcus aureus pneumonia following antimicrobial therapy. *FEMS Immunol. Med. Microbiol.*
**2012**, *65*, 309–317.
11. Aguilera, X.E.; Li Bassi, G.; Wyncoll, D.; Ntoumenopoulos, G.; Fernandez-Barat, L.; Marti, J.D.; Comaru, T.; De Rosa, F.; Rigol, M.; Rinaudo, M.; et al. Tracheal tube biofilm removal through a novel closed-suctioning system: an experimental study. *Br. J. Anaesth.*
**2015**, *115*, 775–783.
12. Li Bassi, G.; Fernandez-Barat, L.; Saucedo, L.; Giunta, V.; Marti, J.D.; Tavares Ranzani, O.; Aguilera Xiol, E.; Rigol, M.; Roca, I. Endotracheal tube biofilm translocation in the lateral Trendelenburg position. *Crit. Care*
**2015**, *19*, 59.
13. Fernandez-Barat, L.; Motos, A.; Panigada, M.; Álvarez-Lerma, F.; Viña, L.; Lopez-Aladid, R.; Ceccato, A.; Bassi, G.L.; Nicolau, D.P.; Lopez, Y.; et al. Comparative efficacy of linezolid and vancomycin for endotracheal tube MRSA biofilms from ICU patients. *Crit. Care*
**2019**, *23*, 251.

#### **2.5.5. Aspiration Pneumonia** 

NiedermanMichael S.Clinical Director and Associate Chief, Pulmonary and Critical Care Medicine, Weill Cornell Medical Center, New York, NY, USA

Aspiration pneumonia can occur in patients both inside and out of the hospital, and can lead to severe illness and respiratory failure. 

The term “aspiration pneumonia” is used to refer to macroaspiration of the contents of a colonized oropharynx or stomach. When an aspiration event occurs, it can be of bacteria, gastric acid or inert material (eg. blood). When bacteria are aspirated, the development of pneumonia depends on the virulence of the organisms, the size of the inoculum, and the state of the patient’s host defenses. The identity of the etiologic organisms depends on the site of acquisition (home, hospital, etc.). Aspiration pneumonia not only occurs in patients with appropriate risk factors (altered consciousness, protracted vomiting, mechanical disruption of defense barriers, sedating medications, impaired cough and swallowing or gastric problems) but occurs in characteristic anatomic locations, primarily gravity-dependent regions of the lung such as the lower lobes or posterior upper lobe, and more on the right side than the left. In the ICU, as many as 20% of patients have dysphagia and aspiration post extubation and many of them continue to aspirate at the time of hospital discharge. Also in the ICU, patients who have hypothermia therapy after cardiac arrest are especially at risk for aspiration pneumonia, within 2–4 days post event, since hypothermia can impair host defenses, and urgent intubation post arrest can promote the entry of oral contents into the lungs. 

The bacteriology of aspiration pneumonia has changed from years past, with anaerobes being relatively uncommon except in patients with poor dentition and impaired consciousness. Gram-negative bacteria are more common than other organisms, in patients who develop pneumonia in a hospital or chronic care facility. Clinically patients with aspiration events do not always have a witnessed event, so suspicion should be high in those with risk factors. Conversely, not every witnessed aspiration event leads to pneumonia and if aspiration leads to symptoms, they can include airway findings such as cough and bronchospasm, or parenchymal lung findings such as hypoxemia, dyspnea and respiratory failure. Up to 16% of aspiration pneumonia can lead to ARDS, but the risk rises if other ARDS risks are also present. 

The diagnosis of aspiration pneumonia is made by a constellation of clinical features, risk factors and radiographic findings. In the differential diagnosis is bacterial pneumonia, chemical pneumonitis, foreign body obstruction with distal pneumonia, and negative pressure pulmonary edema, the latter in patients undergoing intubation and general anesthesia. Biomarkers such as procalcitonin cannot be relied on to distinguish chemical from bacterial pneumonitis. 

An algorithm for therapy will be presented that differs for patients with community acquired or nosocomial aspiration, because each has different colonizing bacteria. Although the use of antibiotics is usually driven by the presence of radiographic abnormalities, some patients with severe illness after a witnessed aspiration episode are also treated with antibiotics, because of the possibility of delayed appearance of radiographic infiltrates. Duration of therapy is dictated by clinical response, but can be as short as 5 days, and can be guided by serial measurements of procalcitonin. 

Prevention is key for patients at risk for aspiration. Key interventions include: no food for 8 h and no clear liquids for at least 2 h prior to elective surgery; 24 h of prophylactic antibiotics following urgent intubation, especially in those with impaired consciousness, including those treated with therapeutic hypothermia post cardiac arrest; swallowing evaluation after stroke and post extubation; feeding in a semi-recumbent position post stroke; and preference for an angiotensin –converting enzyme inhibitor to treat hypertension post stroke (since it can promote cough). For patients with impaired consciousness in a long term care facility, oral care has uncertain benefit, and the use of oral chlorhexidine remains controversial. 

##### **References** 

1. Mandell, L.A.; Niederman, M.S. Aspiraton pneumonia. *N. Engl. J. Med*. **2019**, *380*, 651–663.
2. El-Solh, A.A.; Pietrantoni, C.; Bhat, A.; Okada, M.; Zambon, J.; Aquilina, A.; Berbary, E. Colonization of dental plaques: a reservoir of respiratory pathogens for hospital acquired pneumonia in institutionalized elders. *Chest*
**2004**, *126*, 1575–1582.
3. El-Solh, A.A.; Pietrantoni, C.; Bhat, A.; Aquilina, A.T.; Okada, M.; Grover, V.; Gifford, N. Microbiology of Severe Aspiration Pneumonia in Institutionalized Elderly. *Am. J. Respir. Crit. Care Med.*
**2003**, *167*, 1650–1654.
4. Perbet, S.; Mongardon, N.; Dumas, F.; Burel, C.; Lemiale, V.; Mourvillier, B.; Carli, P.; Varenne, O.; Mira J.P.; Wolff, M.; et al. Early-onset pneumonia after cardiac arrest: characteristics, risk factors and influence on prognosis. *Am. J. Respir. Crit. Care Med*. **2011**, *184*, 1048–1054.
5. Vallès, J.; Peredo, R.; Burgueño, M.J.; De Freitas, A.P.R.; Millan, S.; Espasa, M.; Martin-Loeches, I.; Ferrer, R.; Suarez, D.; Artigas, A. Efficacy of Single-Dose Antibiotic Against Early-Onset Pneumonia in Comatose Patients Who Are Ventilated. *Chest*
**2013**, *143*, 1219–1225.
6. Shinohara, Y.; Origasa, H. Post-Stroke Pneumonia Prevention by Angiotensin-Converting Enzyme Inhibitors: Results of a Meta-analysis of Five Studies in Asians. *Adv. Ther.*
**2012**, *29*, 900–912.

### 2.6. SESION VI: Diagnostic Tools in Pulmonary Infection

#### **2.6.1. New Diagnostic Approaches for Ventilator-Associated Pneumonia** 

ChastreJeanFrom the Service de Médecine Intensive − Réanimation, Institut de Cardiométabolisme et Nutrition (iCAN), Hôpital La Pitié–Salpêtrière, Sorbonne Université, Assistance Publique–Hôpitaux de Paris (APHP), Paris, France.Ventilator-associated pneumonia (VAP) is typically identified at the bedside by combining three criteria: (1) clinical observations suggesting infection, e.g., the new onset of fever, purulent sputum, leukocytosis, increased minute ventilation, arterial oxygenation decline and/or the need for increased vasopressor infusion to maintain blood pressure; (2) new or progressive persistent radiographic infiltrates; and (3) “positive” microbiological culture results for a potentially pathogenic microorganism isolated from endotracheal aspirates (ETAs), bronchoalveolar lavage (BAL) fluid, pleural fluid and/or blood.^1^ However, this VAP-case definition is frequently inaccurate and leaves room for subjective interpretation.^2^ Since a new pulmonary infiltrate is the only criterion confirming involvement of the intra-alveolar pulmonary spaces by the infectious process, it is considered a prerequisite for the diagnosis of VAP. However, most of the time it is not present, with the infection remaining confined to an already abnormal region of the lung, rendering useless all diagnostic approached based on lung sonography and radiology, including CT scans.^3^ Indeed, most ICU patients, if not all, demonstrate bi-basal pulmonary infiltrates when screen with CT scan or lung sonography after ≥2 days of mechanical ventilation (MV).^4^ Thus, it is mandatory to consider the diagnosis of VAP in patients who deteriorate clinically, and/or in whom vasopressors need to be increased in order to maintain blood pressure, even in the absence of a clear-cut progression of radiographic abnormalities. The presence of a pathogen at the level of the proximal airways does not suffice for making the diagnosis of VAP, since most ventilated patients are colonized with pathogens very early after the initiation of MV. Even deciding which threshold should be applied to define a “positive” culture when using semi-quantitative or quantitative ETA or BAL fluid cultures can be problematic, especially for specimens obtained after starting new antibiotics.^3^ Thus, the absence of undisputable “reference standards” continues to fuel controversy about the adequacy and relevance of many studies in this field and have led investigators to describe either other types of lower respiratory tract infection, e.g., ventilator-associated tracheobronchitis (VAT), or even to abandon the concept of VAP and replace it by a new construct that comprises different levels of “ventilator-associated events”, including infection-related ventilator-associated condition (IVAC).^5^ These current limitations in establishing a rapid and precise microbiologically confirmed diagnosis of VAP serve as the impetus for developing new rapid diagnostic approaches for this important infection. Multiplex real-time PCR, peptide nucleic acid fluorescence in-situ hybridization (PNA-FISH) and matrix-assisted laser desorption ionization time-of-flight mass spectrometry (MALDI-TOF MS) are all very accurate techniques that allow rapid detection of a broad array of respiratory pathogens to optimize empiric antimicrobial treatment, including bacteria, yeast, mold, viruses, and mycobacteria.^6,7^ These platforms are now marketed by different companies and can be used in many microbiological laboratories in a routine manner. The benefits to this can be numerous, including potential for providing timely administration of appropriate antimicrobial therapy as well as minimizing the use of broad-spectrum antibiotics when they are not justified. They can also facilitate clinical trials for new anti-infective agents by stratifying patients eligible for the trial at the earliest possible opportunity.^8^ However, it is also important to understand the limitations of these new technologies. Although some of them, including quantitative PCR (qPCR), allow determination of the absolute quantity of target DNA in a sample according to a calibration curve, they cannot distinguish between live and dead cells. Therefore, they could grossly overestimate the bacterial burden present in the specimens. As such, they have an inherent limited capacity for differentiating colonization and tracheobronchitis from true infection requiring immediate antimicrobial treatment, which could be highly problematic in mechanically-ventilated patients. Secondly, although several genes coding for antimicrobial resistance can be detected by molecular technologies, their mere presence does not automatically mean their expression or production. The latter is true with the exception of a few specific mechanisms of resistance provided by the above described molecular techniques (i.e., detection of methicillin-resistant *Staphylococcus aureus*) and potentially the Accelerate Pheno^TM^ System, which is the only FDA cleared platform currently available that provides both pathogen identification and phenotypic antimicrobial susceptibility using morphokinetic cellular analysis using advanced optics microscopy.^9^ Therefore, although typing tests based on qPCR and other molecular diagnostic methods are faster, their results regarding the true susceptibility patterns of the causative pathogens should always be correlated with phenotypic and biochemical tests. As such, it is usually not possible to de-escalate from broad-spectrum antimicrobial coverage to a more narrow-spectrum regimen before a conventional antibiogram is obtained. Thirdly, the deployment of these new molecular techniques is not easy and should always be done in close collaboration with ICU doctors, embedded in a strong antibiotic stewardship program in order to reduce the time to appropriate antimicrobial therapy in patients infected with MDR pathogens, as well as avoiding broad-spectrum regimens when the infection can be treated by a narrow-spectrum antibiotic. 

##### **References** 

1. Torres, A.; Niederman, M.S.; Chastre, J.; Ewig, S.; Fernandez-Vandellos, P.; Hanberger, H.; Kollef, M.; Li Bassi, G.; Luna, C.M.; Martin-Loeches, I.; et al. International ERS/ESICM/ESCMID/ALAT guidelines for the management of hospital-acquired pneumonia and ventilator-associated pneumonia: Guidelines for the management of hospital-acquired pneumonia (HAP)/ventilator-associated pneumonia (VAP) of the European Respiratory Society (ERS), European Society of Intensive Care Medicine (ESICM), European Society of Clinical Microbiology and Infectious Diseases (ESCMID) and Asociacion Latinoamericana del Torax (ALAT). *Eur. Respir. J*. **2017**, *50*, pii1700582.
2. Klompas, M. Does This Patient Have Ventilator-Associated Pneumonia? *JAMA*
**2007**, *297*, 1583.
3. Chastre, J.; Luyt, C.-E. Does this patient have VAP? *Intensiv. Care Med.*
**2016**, *42*, 1159–1163.
4. Bouhemad, B.; Dransart-Rayé, O.; Mojoli, F.; Mongodi, S. Lung ultrasound for diagnosis and monitoring of ventilator-associated pneumonia. *Ann. Transl. Med*. **2018**, *6*, 418.
5. Martin-Loeches, I.; Póvoa, P.; Rodríguez, A.; Curcio, D.; Suarez, D.; Mira, J.-P.; Cordero, M.L.; LePecq, R.; Girault, C.; Candeias, C.; et al. Incidence and prognosis of ventilator-associated tracheobronchitis (TAVeM): a multicentre, prospective, observational study. *Lancet Respir. Med.*
**2015**, *3*, 859–868.
6. Zumla, A.; A Al-Tawfiq, J.; I Enne, V.; Kidd, M.; Drosten, C.; Breuer, J.; A Müller, M.; Hui, D.; Maeurer, M.; Bates, M.; et al. Rapid point of care diagnostic tests for viral and bacterial respiratory tract infections—needs, advances, and future prospects. *Lancet Infect. Dis.*
**2014**, *14*, 1123–1135.
7. Timbrook, T.T.; Spivak, E.S.; Hanson, K.E. Current and Future Opportunities for Rapid Diagnostics in Antimicrobial Stewardship. *Med Clin. N. Am.*
**2018**, *102*, 899–911.
8. Guillamet, M.C.V.; Burnham, J.P.; Kollef, M.H. Novel Approaches to Hasten Detection of Pathogens and Antimicrobial Resistance in the Intensive Care Unit. *Semin. Respir. Crit. Care Med.*
**2019**, *40*, 454–464.
9. Descours, G.; Desmurs, L.; Hoang, T.L.T.; Ibranosyan, M.; Baume, M.; Ranc, A.G.; Fuhrmann, C.; Dauwalder, O.; Salka, W.; et al. Evaluation of the Accelerate Pheno™ system for rapid identification and antimicrobial susceptibility testing of Gram-negative bacteria in bloodstream infections. *Eur. J. Clin. Microbiol. Infect. Dis*. **2018**, *37*, 1573–1583.
10. Timbrook, T.T.; Morton, J.B.; McConeghy, K.W.; Caffrey, A.R.; Mylonakis, E.; LaPlante, K.L. The effect of molecular rapid diagnostic testing on clinical outcomes in bloodstream infections: a systematic review and meta-analysis. Clin. Infect. Dis. **2017**, *64*, 15–23.

#### **2.6.2. New Sensing Nanodevices for Early Diagnosis of Infectious at the Point-of-Care** 

LechugaLaura M.Nanobiosensors and Bioanalytical Applications Group, Catalan Institute of Nanoscience and Nanotechnology (ICN2), CSIC, BIST and CIBER-BBN. Campus UAB, 08193 Barcelona, Spain

Infections and multidrug-resistance have become a major healthcare issue in the 21st Century with millions of cases every year and with an increasing incidence of deaths. In Europe 5–10% of all hospitalizations results in nosocomial infections, with the highest rates in surgical and intensive care units (ICU) [1,2]. Diagnosis and management of infections in hospital settings is a very critical task where fast and accurate results can translate into life, changing health outcomes for individuals. This is especially severe in the case of nosocomial infections, and when the infections evolves in sepsis. In particular, sepsis with a mortality rate of ~30% requires fast and accurate diagnosis as the survival chances decreases by 7–8% for every hour that the infection remains untreated [3]. Rapid, sensitive and quantifiable bacterial detection from patient blood is thus a clinical demand. Despite the advances in the conventional diagnostic techniques, these are inherently time consuming and labour intensive and antimicrobial therapy can render negative cultures, thus slowing the finding of the right treatment. Thus, diagnostic tools with enhanced sensitivity and selectivity, able to prompt identify the type of infection and the bacteria resistance profile could greatly help in the administration of the most suitable treatment, improving the patient’s outcome.

We work in developing such clinical point-of-care (PoC) devices by employing novel nanophotonic biosensor platforms as a powerful tool for rapid infection detection and for the identification of the genetic sequences associated to the antibiotic susceptibility. We have demonstrated, at laboratory level, that our biosensor technology is suitable for the rapid and label-free detection of spontaneous bacterial peritonitis. The biosensor device is capable of detecting *E. coli* at extremely low concentrations (LOD: 4 cfu/mL) in human ascitic fluid, using a label-free direct immunoassay, with a time to result of only 25 min and without the need of any previous sample purification [4]. Moreover, we have developed an ultrasensitive biosensor for the detection of genes associated with the multidrug-resistance found in Gram-negative bacteria (as *E. coli*) without the need of any PCR amplification step [5]. We have detected genes encoding several β-lactamase enzymes able to hydrolyze a broad spectrum of beta-lactams. As a proof-of-concept, we detected two genes of the unamplified genomic DNA directly extracted from bacteria commonly found in real samples of patients attended at Vall d’Hebron Hospital. All the steps took 30 min, achieving a LOD of only 5.8 aM (~10^5^ copies) [5]. This value represents one of the most sensitive DNA detection without amplification or labeling steps reported up to date, and demonstrates the excellent potential of our nanophotonic biosensor technology for the identification of superbug genes. 

Moreover, we have accomplished steps towards an autonomous and portable sensing platform for providing a label-free methodology in the clinical practice, reducing the sample volume and the time-to-result. This compact POC biosensor device has been employed for the detection of sepsis biomarkers (bacteria, proteins and microRNAs) from real patient blood samples at Vall d’Hebron Hospital. Our device operates with low sample volumes (10 µL) and is a quick (40 min) one-step quantification method without the need of multiple expensive laboratory instruments, reagents or skilled technicians, thereby offering a user-friendly fast and sensitive clinical PoC device. When employed in the hospital, the biosensor device has enabled accurate categorization of sepsis patients (infectious SIRS) from control groups (healthy individuals and non-infectious SIRS patients) without false positives/negatives [6,7].

Our approach paves the way for modern implementable PoC diagnostics in the clinical settings for infectious detection. The biosensor POC device could be employed by non-expert personnel at the bed-side of patients and could has a strong impact in guiding quick medical decisions across various clinical scenarios.

##### **References** 

1. Brusaferro, S.; Arnoldo, L.; Cattani, G.; Fabbro, E.; Cookson, B.; Gallagher, R.; Hartemann, P.; Holt, J.; Kalenic, S.; Popp, W.; et al. Harmonizing and supporting infection control training in Europe. *J. Hosp. Infect.*
**2015**, *89*, 351–356.
2. Garcia, I.J.; Torné, E.E.; Arriortua, A.B.; Vicente, J.C.D.C.; Soler, P.G.; Torre, J.A.C.; González, J.C.F.; Revilla, P.M.; Martínez, M.P.; VINCIP Study Group, from Spanish Society of Pediatric Intensive Care (SECIP) Trends in nosocomial infections and multidrug-resistant microorganisms in Spanish pediatric intensive care units. *Enfermedades Infecciosas y Microbiología Clínica*
**2016**, *34*, 286–292.
3. Levy, M.M.; Artigas, A.; Phillips, G.S.; Rhodes, A.; Beale, R.; Osborn, T.; Vincent, J.-L.; Townsend, S.; Lemeshow, S.; Dellinger, R.P. Outcomes of the Surviving Sepsis Campaign in intensive care units in the USA and Europe: a prospective cohort study. *Lancet Infect. Dis.*
**2012**, *12*, 919–924.
4. Maldonado, J.; González-Guerrero, A.B.; Domínguez, C.; Lechuga, L.M. Label-free bimodal waveguide immunosensor for rapid diagnosis of bacterial infections in cirrhotic patients. *Biosens. Bioelectron.*
**2016**, *85*, 310–316.
5. Maldonado, J. Interferometric Biosensors for Rapid Identification of Nosocomial Infections. Ph.D. Thesis, Univ. Autónoma de Barcelona. 2017. Available online: https://ddd.uab.cat/record/180104.
6. Dey, P.; Fabri-Faja, N.; Calvo-Lozano, O.; Terborg, R.A.; Belushkin, A.; Yesilköy, F.; Fàbrega, A.; Ruiz-Rodriguez, J.C.; Ferrer, R.; González-López, J.J.; et al. Label-free Bacteria Quantification in Blood Plasma by a Bioprinted Microarray Based Interferometric Point-of-Care Device. *ACS Sensors*
**2018**, *4*, 52–60.
7. Fabri-Faja, N.; Calvo-Lozano, O.; Dey, P.; Terborg, R.A.; Estévez, M.-C.; Yesilköy, F.; Pello, J.; Altug, H.; Pruneri, V.; Lechuga, L.M. Detection of protein and miRNA biomarkers for early Sepsis diagnosis with an optical Point-of-care microarray biosensor. *Ann. Clinica Acta*
**2019**, *1077*, 232–242.

### 2.7. SESION VII: Optimizing Antimicrobial Therapy and Prevention

#### **2.7.1. Prevention of VAP: How We Made Progress. The ANTHARTIC trial** 

FrancoisB.CHU Limoges and France

Ventilator-Associated Pneumonia remains the most important infectious complication in the ICU resulting in prolongation of hospitalization, potentially increasing the risk of death but also being the main driver of antibiotic consumption in critically ill patients. When considering the antimicrobial resistance threat, reduction of antibiotic use is key and therefore prevention strategies against VAP should be promoted. Within this field, very few approaches have shown efficacy and currently outside the classical VAP bundles, no specific treatment is fully recommended and demonstrated. Both chlorhexidine, SOD/SDD and probiotics have failed to truly confirm a beneficial effect. In this field, monoclonal antibodies could represent an alternative option in VAP prevention with some promising results against Staphylococcal VAP but are still in early clinical development. In fact, even if not fully disruptive, the only promising strategy that have shown benefit is based on short-term prophylactic antibiotics. Most of the what has been positively evidenced has been done in neuro or comatose patients and mostly using cephalosporin. In general, the benefit is on VAP incidence and ICU stay without significant reduction of mortality which remain mostly driven by the underlying diseases. Patients treated with hypothermic Targeted Temperature Management (TTM), which is a standard of care after out-of-hospital cardiac arrest (OHCA) with shockable rhythm are one of the most at risk population for VAP during ICU stay, up to 60%, and therefore to be consider strongly for VAP prevention. Nevertheless, the benefit of a preventive short-term antibiotic therapy has not yet been shown prospectively in such a population. In most cardiac arrest studies, susceptible gram positive cocci and *Haemophilus influenzae* are the more common bacteriae documented in early VAP. Therefore, amoxicillin-clavulanic acid appears to be the most appropriate antibiotic to be used, since its spectrum encompasses targeted bacteriae with a moderate selection pressure and acceptable cost. In contrast, ceftriaxone or other cephalosporins which has been used in some trials has a broader spectrum with associated greater selection pressure, but a lower activity on MSSA which need to be targeted in the OHCA population, at least in Europe. To hypothesize the crude reduction for our study, we have considered the results from the three available RCT dealing with early-onset VAP and based on these results, we have decided that a 25% crude reduction would be meaningful and achievable.

The ANTHARTIC study is a multicentre, placebo-controlled, randomized trial which have enrolled ICU patients >18 years, mechanically ventilated after OHCA related to initial shockable rhythm and treated with 32–34 °C-TTM. Patients with extracorporeal life supports, ongoing antibiotic therapy, chronic colonization with multiresistant bacteria, allergy to beta-lactam antibiotics or with moribund status were excluded. Either IV injection of amoxicillin-clavulanic acid (1 g/200 mg) or placebo was administered 3 times a day for 2 days, starting less than six hours after the OHCA. Primary outcome was early VAP (≤7 days) which was assessed by an independent adjudication committee based on pre-defined strict clinical, radiological and microbiological criteria using widely accepted criteria derived from 2010 FDA guidance. Health economic evaluation was performed during initial admission and 12-month readmissions and MDR screening was done at admission and at D7.

198 patients were randomized and 194 were analysed. Patient care was well standardized across participating sites not only sharing exactly the same healthcare system but also more specifically when considering bundles to prevent VAP for which recommendations were given at time of site initiation. After adjudication, 60 VAP were confirmed including 51 early VAP. Most VAP were polymicrobial (60%) with microbiological documentation in the control group similar to that previously described in European ICUs, with a predominance of Gram-negative bacteria susceptible to amoxicillin-clavulanic acid. Antibiotic prophylaxis significantly decreased the development of early VAP (19 [19.2%] vs. 32 [33.7%]: p = 0.03; HR = 0.53; 95%CI: 0.31–0.92). Similar results were obtained when using the 5-day cut-off value to distinguish early from late VAP (cumulative incidence on day 5: 17.2% in the antibiotic group vs. 30.5% in the control group; HR = 0.53; 95%CI = 0.30; 0.95; p = 0.03). No difference in VAP incidence was evidenced when considering the targeted temperature management method and no centre effect was noted. Overall, the risk of developing VAP was lower in patients who received antibiotics (HR = 0.55; 95%CI = [0.33; 0.91]). Non-pulmonary secondary infectious complications were equally distributed between both study arms. No difference in occurrence of late VAP (4% vs. 5.3%) and Day-28 mortality (41.4% vs. 37.5%) was observed, knowing that mortality were mostly related to cardiac events or care limitations. Reduction of mean total costs reached €7193 per patient in the antibiotic group when considering the overall 12-month follow-up period including initial stay and readmissions. Some authors had suggested that such therapeutic strategy could alter the microbiota and the epidemiology of secondary infections but in our limited sampling we only noted the increased frequency of enterobacteria in patients treated with amoxicillin-clavulanic acid, however, the implications are unclear. No argument supports a potential promotion of difficult-to-treat organisms when using a routine two-day course of amoxicillin-clavulanic acid, which is in keeping with previous studies. None of serious adverse event was considered to be related to study treatment by investigators.

A two-day antibiotic therapy with amoxicillin-clavulanic acid in patients receiving a 32–34 °C-TTM strategy after OHCA with initial shockable rhythm is safe and significantly reduces early VAP and hospital costs. Overall, this antibiotic approach combines lower costs and better outcome making two-day antibiotic therapy a dominant strategy compared to usual care for the prevention of early VAP and therefore could be recommended as standard of care in all OHCA patients receiving TTM. But, whether the present results apply to all OHCA patients, including non-shockable rhythms or managed with a different targeted temperature remains to determine. 

##### **References** 

1. Perbet, S.; Mongardon, N.; Dumas, F.; Bruel, C.; Lemiale, V.; Mourvillier, B.; Carli, P.; Varenne, O.; Mira, J.P.; Wolff, M.; et al. Early-onset pneumonia after cardiac arrest: characteristics, risk factors and influence on prognosis. *Am. J. Respir. Crit. Care Med*. **2011**, *184*, 1048–1054.
2. Davies, K.J.; Walters, J.H.; Kerslake, I.M.; Greenwood, R.; Thomas, M.J. Early antibiotics improve survival following out-of hospital cardiac arrest. *Resusc.*
**2013**, *84*, 616–619.
3. Gagnon, D.J.; Nielsen, N.; Fraser, G.L.; Riker, R.R.; Dziodzio, J.; Sunde, K.; Hovdenes, J.; Stammet, P.; Friberg, H.; Rubertsson, S.; et al. Prophylactic antibiotics are associated with a lower incidence of pneumonia in cardiac arrest survivors treated with targeted temperature management. *Resusc.*
**2015**, *92*, 154–159.
4. Nair, G.B.; Niederman, M.S. Ventilator-associated pneumonia: present understanding and ongoing debates. *Intensive Care Med*. **2015**, *41*, 34–48.
5. Ribaric, S.F.; Turel, M.; Knafelj, R.; Gorjup, V.; Stanic, R.; Gradisek, P.; Cerovic, O.; Mirkovic, T.; Noc, M. Prophylactic versus clinically-driven antibiotics in comatose survivors of out-of-hospital cardiac arrest—A randomized pilot study. *Resusc.*
**2017**, *111*, 103–109.

#### **2.7.2. New Antibiotics for Severe CAP: Ceftaroline** 

TorresAntoniPulmonology Department, Clinic Hospital, University of Barcelona, Barcelona, CIBER Enfermedades Respiratorias, Spain

Ceftaroline is a fifth G Cephalosporin with a good activity against Gram positives including *S. pneumoniae* and *S.aureus MSSA* and *MRSA*. In addition, it has a good activity against *Enterobacteriaceae* non-ESBL. The registrational trials (1,2) comparing 600 mg/12 h IV with 1 g of ceftriaxone in hospitalized CAP demonstrated significant superiority in clinical cure in favor of ceftaroline (3), when pooling together the registrational trial results. In a subsequent randomized study (Asia CAP) using 2 g of ceftriaxone +/− macrolide showed significant higher clinical cure rates of ceftaroline. A real world observational study (4 Capture) showed high rates of clinical cure of patients with CAP with the following characteristics: elderly, patients with comorbidities, patients with renal efailure, *S.pneumoniae, S.aureus MSSA,* and *MRSA.* According to published RCT, the recent ATS/IDSA CAP guidelines (5), recommended ceftaroline as one of the antibiotics to be added to macrolides in the empirical treatment hospitalized CAP.

Severe CAP in patients admitted to the ICU has a high mortality that ranges from 25% to 40% depending on the existence of shock and /or the need for mechanical ventilation (5) (despite initial adequate antibiotic treatment). Any antibiotic that could increase the rates of clinical cure, and eventually mortality, needs to be included in the empirical treatment of SCAP. In one bi-center observational study in SCAP patients (6) we observed a high clinical cure rate. In addition, mortality was significantly associated with the late administration of ceftaroline. In another study (7) using a network meta-analysis, ceftaroline was one of the two antibiotics associated with lower mortality in hospitalized CAP. Although there are not RCT´`s in SCAP comparing ceftaroline to ceftriaxone or cefotaxime, the higher rates of clinical cure, its broad antimicrobial activity including *S.pneumoniae* and *S.aureus MSSA and MRSA,* suggests that ceftaroline should be a first -line antibiotic for SCAP. In patients with MRSA suspicion or with influenza that have a high change to be co-infected with *S.aureus*, ceftaroline is a very good choice as well.

##### **References** 

1. File, T.M. Jr.; Low, D.E.; Eckburg, P.B.; Talbot, G.H.; Friedland, H.D.; Lee, J.; Llorens, L.; Critchley, I.A.; Thye, D.A. FOCUS 1 investigators. FOCUS 1: a randomized, double-blinded, multicentre, Phase III trial of the efficacy and safety of ceftaroline fosamil versus ceftriaxone in community-acquired pneumonia. *J. Antimicrob. Chemother.*
**2011**
*66*, 19–32.
2. Low, D.E.; File, T.M. Jr.; Eckburg, P.B.; Talbot, G.H.; Friedland, D.H.; Lee, J.; Llorens, L.; Critchley, I.A.; Thye, D.A. FOCUS 2 investigators. FOCUS 2: a randomized, double-blinded, multicentre, Phase III trial of the efficacy and safety of ceftaroline fosamil versus ceftriaxone in community-acquired pneumonia. *J. Antimicrob. Chemother*. **2011**, *66*, 33–44.
3. Taboada, M.; Melnick, D.; Iaconis, J.P.; Sun, F.; Zhong, N.S.; File, T.M.; Llorens, L.; Friedland, H.D.; Wilson, D. Ceftaroline fosamil versus ceftriaxone for the treatment of community-acquired pneumonia: individual patient data meta-analysis of randomized controlled trials. *J. Antimicrob. Chemother.*
**2016**, *71*, 862–870.
4. Zhong, N.S.; Sun, T.; Zhuo, C.; D'Souza, G.; Lee, S.H.; Lan, N.H.; Chiang, C.H.; Wilson, D.; Sun, F.; Iaconis, J.; et al. Diagnosis and Treatment of Adults with Community-acquired Pneumonia. An Official Clinical Practice Guideline of the American Thoracic Society and Infectious Diseases Society of America. *Am. J. Respir. Crit. Care Med.*
**2019**, *200*, e45–e67.
5. Bassetti, M.; Russo, A.; Cillóniz, C.; Amaro, R.; Graziano, E.; Soriano, A.; Torres, A. Ceftaroline for Treatment of Severe Pneumonia: A Real-Word multicenter Experience. *J. Infec*. Under Review.
6. Montes-Andújar, L.; Tinoco, E.; Baez-Pravia, O.; Blanco-Schweizer, P.; Segura, C.; Prol Silva, E.; Reyes, V.; Rodríguez-Cobo, A.; Zurdo, C.; Angeles, V.; et al. Empiric antibiotic for Community-acquired pneumonia in adult patient. A network meta-analysis. Under Review.

#### **2.7.3. Intensive Care Units Are Epicentres for Antimicrobial Resistance Development** 

CarletJean^1^De WaeleJan^2^1World Alliance Against Antibiotic Resistance (WAAAR), Creteil, France2University Hospital of Ghent, Ghent, Belgium

Antimicrobial resistance (AMR) is increasing rapidly to a dangerous level worldwide, both in animals and humans. Obviously, we have not been able to protect antibiotics, this precious resource. The environment, both inside and outside the hospitals, is also heavily involved. Effluents coming from the hospitals are heavily colonized with high inoculum of multi-drug resistant micro-organisms (MDRO) and contain high concentrations of antibiotics. Although most of the antibiotics are consumed in the community, the hospitals are a very important in the emergence of antibiotic resistance, both because antibiotics are often over-used, and because MDRO are easily transmitted from patient to patient, via the hands of the health care professionals and equipment used. Intensive care units (ICUs) particularly should be deeply concerned by the issue of AMR.

For many reasons, ICUs are the «epicentres» for the selection and diffusion of MDRO, and as such they have an important role in the emergence of AMR, and therefore also in the prevention thereof. Large scale prevalence studies have shown that up to 70% of ICU patients are treated with antibiotics on a randomly chosen day. Antibiotics used are very often broad-spectrum drugs inducing the likelihood that AMR may develop. The intestinal microbiome including the oropharynx, but mostly the distal gut plays a key role in the «hidden» selection of highly resistant micro-organisms. The very high intensity of care in the ICUs dramatically increases the risk of patient to patient cross-transmission of those bacteria via the hand of the health care professionals, and the environment. This problem is the same in the step-down units, in which the nurse to bed ratio is far lower. Severe infections can occur due to those MDRO, including urinary tract infections, central line infections and ventilator-associated pneumonia. Prolonged, and sometimes repeated antibiotic exposure selects micro-organisms which are increasingly resistant, particularly in the gut and in the ventilated lungs. In some cases, and some countries, severe infections with bacteria resistant to every available antibiotic have been reported. 

Resistant bacteria can easily spread to the other wards of the hospital, to long-term facilities or to the community when the patients are discharged from the ICU, in particular if the colonization of the patient with resistant bacteria is not properly notified to the new caretaker, the general practitioner or outpatient nursing team.

For all the above reasons, the ICU physicians and nurses have a very special responsibility in the management of antibiotics and the prevention of development and spread of AMR.

It is very clear that AMR is consistently increasing in the ICUs worldwide. There are huge differences between countries, regions, hospitals or even ICUs within the same facility. In a multicentre European study on 37 ICUs, the prevalence of MRSA ranged from 0% up to 100%. Similar differences were noted in this study for ESBL-producing enterobacteriaceae, *Pseudomonas aeruginosa* and *Acinetobacter baumanii*. Northern-European countries like Scandinavian ones, Iceland, the Netherlands, Switzerland, and to a lesser extend Germany historically have low antibiotic consumption and AMR levels. In other countries, like Italy, Greece, India, Asian countries such as China, North Africa and Middle East countries, the level of resistance is very high, with a prevalence of ESBL or carbapenemases above 50%. Those differences are probably mostly due to an over-consumption of antibiotics, particularly broad-spectrum ones, and to poor hygiene practices. However, other unknown factors may play a role.

Gram-negative bacteria resistant to almost every antibiotic are noted more and more frequently in some ICUs, up to as much as a few percent of all pathogens in a Greek study. The development of resistance in *Escherichia coli* to colistin via mcr-1 gene has been an important step in the emergence of those truly omni-resistant bacteria.

There are many ways to limit AMR in the hospitals, in particular in the ICUs and step-down units. The two main actions are antibiotic stewardship, in order to decrease antibiotic consumption and improve antibiotic management, and infection control programs, in order to limit cross-transmission of pathogens. Other actions can be discussed, most of them targeting the digestive microbiome, such as selective digestive decontamination (SDD) with non-absorbable antibiotics for all ventilated patients or just to treat an outbreak due to MDRO, probiotics, bacteriocins, bacteriophages or faecal microbiota transplantation (FMT). Data are still rather scarce to define the efficiency of those recent methods. Those methods to control AMR will be describe in the talk.

##### **References** 

1. Laxminarayan, R.; Duse, A.; Wattal, C.; Zaidi, A.K.; Wertheim, H.F.; Sumpradit, N.; Vlieghe, E.; Hara, G.L.; Gould, I.M.; Goossens, H.; et al. Antibiotic resistance-the need for global solutions. *Lancet Infect. Dis*. **2014**, *14*, 182–196.
2. Carlet, J.; Collignon, P.; Goldmann, D.; Goossens, H.; Gyssens, I.C.; Harbarth, S.; Jarlier, V.; Levy, S.B.; N’Doye, B.; Pittet, D.; et al. Society’s failure to protect a precious resource; antibiotics. *Lancet*
**2011**, *378*, 369–371.
3. Carlet, J. The gut is the epicentre of antibiotic resistance. *Antimicrob. Resist. Infect. Control*
**2012**, *1*, 39.
4. Hanburger, H.; Arman, D.; Gill, H.; Jindrák, V.; Kalenic, S.; Kurcz, A.; Licker, M.; Naaber, P.; Scicluna, E.A.; Vanis, V.; et al., Surveillance of microbial resistance in European intensive care units: a first report from the Care-ICU programme for improved infection control. *Intensive Care Med*. **2009**, *35*, 91–100.
5. Tabah, A.; Koulenti, D.; Laupland, K.; Misset, B.; Valles, J.; Bruzzi de Carvalho, F.; Paiva, J.A.; Cakar, N.; Ma, X.; et al. Characteristics and determinants of outcome of hospital-acquired bloodstream infections in intensive care units: The EUROBACT international cohort study. *Intensive Care Med*. **2012**, *38*, 1930–1945.
6. Yu, H.; Qu, F.; Shan, B.; Huang, B.; Jia, W.; Chen, C.; Li, A.; Miao, M.; Zhang, X.; Bao, C.; et al. Detection of mcr-1 colistin resistance gene in carbapenem-resistant enterobacteriaceae from different hospitals in China. *Antimicrob. Agents Chemother*. **2016**, *60*, 5033–5035.
7. Kollef, M.H.; Bassetti, M.; François, B.; Burnham, J.; Dimopoulos, G.; Garnacho-Montero, J.; Lipman, J.; Luyt, C.-E.; Nicolau, D.P.; Postma, M.J.; et al. The intensive care medicine research agenda on multi-resistant bacteria, antibiotics, and stewardship. *Intensive Care Med.*
**2017**, *43*, 1187–1197.
8. Hans, S.; Shannahan, S.; Pellish, R. Faecal microbiota transplant: Treatment options for Clostridium difficile infection in the intensive care unit. *J. Intensive Care Med*. **2016**, *31*, 577–586.

### 2.8. Poster Presentations

#### **2.8.1. Pneumonia in ICU for Enterobacteria Ceae: Comparative Analysis between Resistant and Multisensible Bacterial** 

MolanoDaniel FrancoVillabónMarioGómezMarioBeltránEdgarCifuentesAlejandraRobayoIvanRodríguezDianaOrdoñezSergioIntensive care Department, Fundation University of sciencies of Health, Hospital San Jose. Bogota-Colombia

##### **Introduction** 

Pneumonia is the first cause of infectious etiology with an indication of admission to the ICU. It is associated with high rates of morbidity and mortality. In low proportion, gram-negative bacteria are the cause of such infections, however, an increase has been progressively seen, especially by multidrug-resistant enterobacteriaceae. In this study we describe the causes associated with the infection and prognosis, in patients with gram-negative multiresistant and multisensitive gram-negative enterobacteriaceae, with admission to the intensive care unit.

##### **Methods** 

Retrospective observational analytic study of patients diagnosed with severe pneumonia and admission to the mixed intensive care unit of the San José Hospital in Bogotá—Colombia, for 3 years (2016–2018).

##### **Results** 

During the analyzed period, a total of 53 patients were treated for pneumonia with isolated gram-negative bacilli, with community-acquired pneumonia 18.8%, health-associated pneumonia 13.2% and nosocomial pneumonia 68%. 33 patients had a diagnosis of severe pneumonia due to multidrug-resistant gram negative (MDR) and 20 patients due to multisensitive gram negative (MS). The average age of MDR infection was 66.1 years vs. 59.1 for MS, with an APACHE II of 18.7 for MDR and 15.4 for MS. 65% of patients infected with MDR germs had multiorgan dysfunction. Within the background, 33% of the patients had chronic lung disease without statistical differences between the presence of antimicrobial resistance in the etiology. 100% of patients required invasive mechanical ventilatory support upon admission. Regarding the prognosis, a statistically significant difference was observed for MDR infections, which had longer days of mechanical ventilation 12.6 SD 8.8 vs. MS infections 8.7 SD 5.2. Regarding days of stay in the ICU, MDR infections had 18.1 SD 10 vs. MS infections 15.3 SD 9.3 and in mortality 42.4% for MDR infections vs. 25% for MS enterobacteria. Klebsiella pneumoniae was the most frequent microorganism in the two groups, with a global percentage of 37.3%, followed by Escherichia coli 22.6% and Enterobacter with 19.1%, without statistical differences between the two groups. 67% of the isolates had a profile of resistance to extended spectrum beta-lactamases (ESBL) and 21 with a KPC type carbapenemase resistant pattern.

##### **Conclusions** 

Patients with pneumonia severe with an indication of admission to the ICU for invasive mechanical ventilation support, have a higher severity score and progression to multi-organ dysfunction, as well as a greater number of days of ventilation, days of hospitalization and mortality if the infection is due to multidrug-resistant enterobacteria compared to multisensitive germs. This difference occurs in infections by Klebsiella pneumonia, E. coli and Enterobacter mainly.

**Table 1.** Demographics and characteristics of pneumonia severe for enterobacteriaceae.
**VARIABLE****ENTEROBACTERIACEAE** **n = 53****MULTI-SENSITIVE** **n = 20****MULTI-RESISTANT** **n = 33****P VALUE**AGE-YEARS (SD)63.9 (18.7)59.1 (23.6)66.9 (14.7)**0.01281**GENDER-M (%)66(%)60(%)72(%)0.36812APACHE II (SD)17.05 (6.23)15.4(7.65)18.7(6.54)0.1173MULTIPLE ORGANIC DYSFUNCTION SYNDROME (%)54.733.365.7**0.02202**BACKGROUND



*COPD (%)*333036.30.63836*IMMUNOSUPPRESSION (%)*121590.50286MECHANICAL VENTILATION DAYS (SD)10.6 (7.12)8.7 (5.2)12.6 (8.8)**0.04781**DAY STAY ICU(SD)16.6 (12.1)15.3 (9.3)18.1(10)0.3154MORTALITY (%)35.82542.40.20054PREVIOUS ANTIBIOTIC USE (%)3626430.2113BETALACTAMS (%)558030**0.00042**CARBAPENEM (%)9.51090.90448ETIOLOGY (%)



*Pseudomonas aeruginosa*7.2590.5892*Klebsiella Pneumoniae*37.335.339.30.77182*E coli*22.630.115.10.1902*Enterobacter spp*19.120.218.10.8493*Acinetobacter Baumanni*2.6500.1936*Proteus* spp.11.110.212.10.83366*Serratia* spp.5.1010.20.13888

#### **2.8.2. HSV type 1 Multifocal Infection—Liver Failure and Brain Disfunction in Transplant Setting** 

FerrãoJoana^1^*DuarteTiago^2^DamiãoFilipe^3^SequeiraTânia^2^CardosoFilipe^2^GermanoNuno^2^1Centro Hospitalar Universitário Lisboa Central, Serviço de Medicina 2.32Centro Hospitalar Universitário Lisboa Central, Unidade de Cuidados Intensivos Polivalente 73Centro Hospitalar Lisboa Norte, Serviço de Gastrenterologia*Correspondence: joanabrancoferrao@gmail.com

Herpes simplex virus (HSV) infections are very common affecting up to 80% of adults during their lifetime with a wide range of clinical manifestations. Most infections are asymptomatic or cause a wide variety of illnesses, most commonly mucocutaneous infections. HVS infection is an uncommon cause of hepatitis and acute liver failure (ALF). The majority of the cases are reported in immunocompromised patients and pregnant women. In immunocompetent patients HVS hepatitis is a particularly rare condition, with few reported cases, and progression to ALF in 74% of the cases with a mortality rate up to 90%. 

We present the case of a 34-year-old male, without prior medical history, presented to the emergency room with fever, abdominal pain and diarrhea for three days. Fever with a maximum peak of 40 °C and abdominal generalized pain, moderate intensity, without relieving or exacerbating factors were the first symptoms. On a first approach with altered liver function tests (AST 1872 U/L, ALT 954 U/L) with mild hyperbilirubinemia (2 mg/dL), leucopenia (3540 cells/uL), decreased platelet count (107,000 cells/uL), CRP 200 mg/L, creatinine 1.5 mg/dL, hyponatremia 121 mmol/L, LDH and clinical signs of dehydration, he was started on IV fluids and empiric antibiotics while the etiologic study was ongoing. Despite adequate medical approach the patient progresses poorly with an episode of generalized tonic clonic seizure on post ictal period, despite no apparent focal neurological deficits, the patient remained with GCS score <8 with the need for orotracheal intubation and transfer to the intensive care unit. 

Further exams were pursued namely a lumbar puncture, with isolation of HSV DNA through PCR reaction and also confirmatory serum HSV DNA. Despite adequate antiviral therapy with acyclovir, clinical and analitical worsening occurred meeting criteria for ALF with a SOFA score over 15. 

Optic nerve sheath diameter (ONSD) on ultrasound evaluation was <5 mm, thus indicating low risk of intracranial hypertension. However, progressive shock with multiple organ failure persisted, with no response to organ-specific supportive care measures. Moreover, after sedation interruption, the patient maintained a GCS of 3 with minor pupillary reflex, no corneal or deep tracheal reflexes and bispectral monitoring of 10. 

Severe brain dysfunction, presumably associated with HSV infection more than with ALF was considered to be a contra-indication for orthotopic liver with severe brain dysfunction, presumably associated with HSV infection more than with ALF was considered to be a contra-indication for orthotopic liver transplantation (OLT) (the patient was unstable to go for brain MRI). The patient died on the 10th day of hospitalization. 

This case illustrates the challenge of managing such patients: How should we face OLT indication, in the presence of a neurological condition with such a poor prognosis? Highvolume plasma exchange can play a role on increasing liver transplant-free survival, but no studies were published regarding specifically ALF with severe encephalitis.(15) Based on the high mortality rates in untreated patients and frequently delayed diagnosis, a high index of suspicion is needed and early diagnosis should be promoted between clinicians; which raises the question: should we start thinking of routinely performing lumbar puncture in patients with ALF, coma and clonus?

#### **2.8.3. Bloody Stenotrophomonas Maltophilia: A Case Series** 

KaklauskaitėJustina^1^JudickasŠarūnas^2^,^3^ŠipylaitėJūratė^2^,^3^1Faculty of Medicine, Vilnius University. Vilnius, Lithuania2Institute of Clinical Medicine, Faculty of Medicine, Vilnius University. Vilnius, Lithuania3Vilnius University Hospital Santaros Klinikos, Centre of Anaesthesiology, Intensive Care and Pain Management. Vilnius, Lithuania

##### **Introduction** 

Stenotrophomonas maltophilia (SM) is commonly a non-virulent, but an important Gram-negative nosocomial pathogen, which can cause various complications. The most vulnerable group is immunocompromised patients, specifically with haematological malignancy and neutropenia, prolonged hospitalization, pneumonia and prior treatment with broad-spectrum antibiotics. SM infection is associated with high morbidity and mortality. 

##### **Objectives** 

To analyze three cases of S. maltophilia caused hemorrhagic pneumonia.

##### **Case Reports** 

Case #1:

A 25-year-old male was diagnosed with acute myeloid leukemia (AML). The patient was treated using two different chemotherapy protocols. Chemotherapy was complicated by febrile neutropenia. The patient received broad spectrum antibiotic therapy. On the 36th day of hospitalization patient developed acute respiratory failure, followed by active pulmonary bleeding. He was admitted to an intensive care unit (ICU). The patient’s status was deteriorating; he was intubated. Diffuse bleeding from both lungs was observed during bronchoscopy. Multiple organ dysfunction progressed and after an unsuccessful cardiopulmonary resuscitation (CPR), the patient died. A day prior the pulmonary bleeding taken blood culture revealed Gram-negative rods that were later identified as multi-drug resistant SM.

Case #2:

A 61-year-old man was diagnosed with acute myeloid leukemia (AML). Chemotherapy did not reach remission. Complete blood count (CBC) showed absolute neutropenia. The patient received broad spectrum antibiotics. On the 45th day of hospitalization, the patient was presented with orthopnea, acute respiratory failure and saturation of 65%, malaise. The patient has caught up bloody sputum a few times and was admitted to the ICU. Respiratory failure progressed and he was intubated. Within a few hours he developed major pulmonary bleeding. A bronchoscopy was performed and bleeding from bronchi of right middle and lower lobes was visible, a balloon obturation of the right intermediate bronchus was performed before an embolization of bronchial artery for right middle and lower lobes. The bleeding continued, refractory shock progressed and after an unsuccessful CPR, the patient died.

Case #3

A 75-year-old man with a history of acute myeloid leukemia (AML) diagnosed two months ago. He received chemotherapy, but no remission was achieved. The patient presented with fever (up to 38.5 °C) and pneumonia. Sputum culture reveled methicillin resistant Staphylococcus haemolyticus. CBC revealed neutropenia. Respiratory dysfunction evolved, he was transferred to the ICU and was intubated. Bronchoscopy was performed. Post-contact mucosal bleeding of trachea and bronchi, blood in bronchoalveolar lavage was observed. Bronchoalveolar aspirate was positive for multidrug-resistant Pseudomonas spp. and SM. The patient was treated with TXM-SMX. Nevertheless, septic shock, pulmonary hemorrhage and multiple organ dysfunction progressed. After an unsuccessful CPR, the patient died.


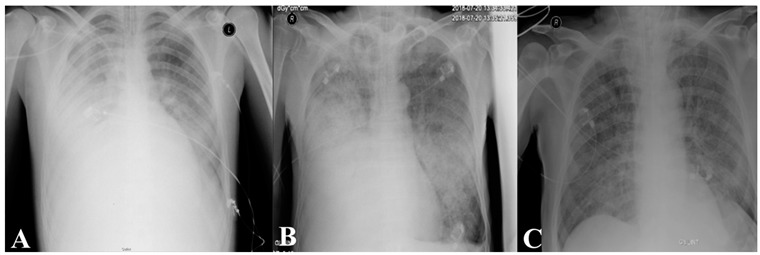


**Figure 1.** Final X-rays of the patients: A—1st case; B—2nd case; C—3rd case.

##### **Discussion** 

Hemorrhagic pneumonia is a rare presentation of SM, and our observed mortality rate in the presented case series was 100% within 12 h after an onset of pulmonary bleeding. All S. maltophilia were susceptible for trimethoprim/sulfamethoxazole. We found that invasive SM infection is lethal for immunocompromised patients but so far there is no evidence about the risk of SM colonization. It is unclear if all the patients positive for SM should receive treatment for SM. 

##### **Conclusions** 

Immunocompromised patients presenting with respiratory failure and signs of pulmonary bleeding should be suspected to have SM infection and early treatment with trimethoprim/sulfamethoxazole should be considered. 

#### **2.8.4. Molecular Characterization of Staphylococcus Aureus Clnical Strains from Endotracheal Tubes of Patients with ICU-Acquired Pneumonia** 

R.Cabrera^1^,^2^L.Fernández-Barat^1^,^2^A.Motos^1^,^2^R.Lopez-Aladid^1^,^2^N.Vázquez^1^,^2^MPanigada^3^F.Álvarez-Lerma^4^A.Ceccato^1^,^2^Y.Lopez^5^L.Viña^6^G.Li Bassi^1^,^2^L.Muñoz^5^T.Israel^2^P.Castro^7^J.M.Nicolas^7^E.Zavala^8^J.Fernandez^9^I.Rovira^10^J.Vila^7^M.Ferrer^1^,^2^A.Torres^1^,^2^1Cellex laboratory, CibeRes ((Centro de Investigación Biomédica en Red de Enfermedades Respiratorias, 06/06/0028)- Institut d’Investigacions Biomèdiques August Pi i Sunyer (IDIBAPS), School of Medicine, University of Barcelona, Spain2Respiratory Intensive Care Unit Pneumology Department, Hospital Clínic, Barcelona, Spain3Department of Anesthesiology, Intensive Care and Emergency, U.O.C. Rianimazione e Terapia Intensiva, Fondazione IRCCS Ca' Granda, Ospedale Maggiore Policlinico, Milan, Italy4Critical Care Dept, Hospital del Mar, Critical Illness Research Group (GREPAC), Hospital del Mar Medical Research Institute (IMIM), Barcelona, Spain5Microbiology Department, Hospital Clínic, CRESIB ISglobal, Barcelona, Spain6Servicio de Medicina Intensiva, Hospital Universitario Central de Asturias, Oviedo, Spain7Medical Intensive care Unit, Hospital Clínic, Barcelona, Spain8Surgical Intensive care Unit, Hospital Clínic, Barcelona, Spain9Hepatic Intensive care Unit, Hospital Clínic, Barcelona, Spain10Cardiovascular Intensive care Unit, Hospital Clínic, Barcelona, Spain

##### **Background** 

*Staphylococcus aureus* is among the most frequently isolated microorganism responsible for ICU-acquired pneumonia of which 29% are resistant to methicillin (MRSA). Our aim was to determine the antimicrobial susceptibility, the associated molecular mechanisms of resistance and the epidemiology relatedness of *S. aureus* strains from the endotracheal tubes (ETT) of intubated critically ill patients with *S.aureus* intensive care unit (ICU) acquired pneumonia.

##### **Materials and Methods** 

Clinical *S. aureus* (17 MRSA and 3 methicillin susceptible *S. aureus*) were collected from ETTs after extubation during a prospective observational study carried out in four European tertiary hospitals. Antimicrobial susceptibility test, using the Kirby-Bauer method was performed to vancomycin, linezolid, ciprofloxacin, clindamycin, erythromycin, chloramphenicol, fusidic acid, gentamicin, quinupristin-dalfopristin, rifampicin, Sulfamethoxazole/trimethoprim, and tetracycline. Interpretation of results was carried out according to the European Committee on Antimicrobial Susceptibility Testing (EUCAST). Molecular characterization of each resistance mechanism was screened by PCR, electrophoresis and sequencing. Molecular epidemiology was analyzed by Multi locus sequence typing. Phylogenetic analysis was carried out using comparative eBURST V3 software (http://www.phyloviz.net/goeburst) 

##### **Results** 

*S. aureus* isolates were resistant to ciprofloxacin (85%), erythromycin (65%), gentamicin (35%), tetracycline (30%), clindamycin (20%), and fusidic acid (5%). Three strains showed hetero-resistant subpopulations to linezolid. The most frequent mutations in ciprofloxacin resistant *S. aureus* strains were S84L in the *gyrA* gene, V511A in the *gyrB* gene, S144P in the *grlA* gene, and K401R/E in the *grlB* gene. Strains resistant to erythromycin carried the *ermC*, *ermA,* and *msrA* genes; the same *ermC* and *ermA* genes were detected in strains resistant to clindamycin. The *aac(6’)-aph(2”)* gene was related to gentamicin resistance, whereas resistance to tetracycline was related to *tetK* (efflux pump). The *fusB* gene was detected in the strain resistant to fusidic acid. The most frequent sequence types were ST22, ST8, and ST217. These were distributed in four clonal complexes (CC5, CC22, CC45, and CC59).

##### **Conclusions** 

High levels of resistance to second-line antimicrobials threatens the treatment of ICU-acquired pneumonia due to methicillin-resistant *S. aureus*, with decreased susceptibility to linezolid and vancomycin. The wide genotypic diversity found, even within the same ICU, reinforces the crucial role of ICU prevention bundles in cross-transmission.

#### **2.8.5. Carbapenem-Resistant Enterobacteriaceae Infection in Subgaleal Space Treated with Meropenem-Vaborbactam: A Case Report** 

ChoiSeohyun (Claudia)^1^,^2^1Ernest Mario School of Pharmacy, Rutgers, The State University of New jersey, USA2Saint Barnabas Medical Center, Livingston, New Jersey, USA

##### **Introduction** 

Carbapenem-resistant *Enterobacteriaceae* (CRE) is an emerging medical challenge worldwide, mainly due to the limitation in numbers of antibiotics that are active against these organisms. Among the different groups of carbapenemase, *Klebsiella pneumoniae* carbapenemase (KPC) is considered to be the most common, but there are others reported as well [1,2]. Current treatment options for CRE infections include polymyxins, tigecycline, fosfomycin, aminoglycosides, ceftazidime-avibactam, meropenem-vaborbactam, or combinations of multiple drugs [3,4]. Even with the available antibiotic options, patients presenting with CRE infections still remain at high risk for mortality [1,5]. 

Meropenem-vaborbactam is a combination product of carbapenem and beta-lactamase inhibitor with Food and Drug Administration (FDA) indication for complicated urinary tract infections including pyelonephritis [6,7]. It showed 99% in vitro activity against *Klebsiella pneumoniae* carbapenemase-producing *Enterobacteriaceae*. Similar to other beta-lactam antibiotics, meropenem-vaborbactam exhibits time-dependent bactericidal activities, and renal excretion is the major pathway of drug clearance [6,7]. 

CRE infections can be associated with various anatomical sites. Common sites of infection reported from our institution are respiratory and urinary tracts. In 2011, there was an outbreak of carbapenemase-producing *Klebsiella pneumoniae* from neurosurgical site infections in China [8]. However, CRE infection associated with central nervous system (CNS) is extremely rare in the United States.

##### **Patient Case** 

Patient was a 69-year-old male with subdural hematoma status post burr hole and craniotomy for re-accumulation. After 2 months, he presented to the hospital with worsening mental status, and CT scan of the head showed sunken craniotomy defect with midline shift, and he underwent for cranioplasty for the defect, which was complicated by uncal herniation. During the post-operative care in the intensive care unit (ICU), patient developed subgaleal fluid collection, which was aspirated. Both subgaleal fluid and blood cultures showed the growth of carbapenem-resistant *Klebsiella pneumoniae*. The cranioplasty implant was removed, and washout and debridement were performed. Patient’s hemodynamic status was stable, not requiring vasopressor support. However, he was minimally responsive throughout the ICU stay. Systemic antibiotic treatment was initiated with ceftazidime-avibactam, which was subsequently switched to meropenem-vaborbactam according to the minimum inhibitory concentration (MIC) result, which were 2 and 0.064, respectively. Due to the inability to administer local antibiotics, patient was managed only with systemic meropenem-vaborbactam 2 g every 12 h intravenously, each dose infused over 3 h. Of note, patient is end-stage renal disease receiving intermittent hemodialysis. Repeat blood cultures remained negative 3 days after the initiation of treatment. Culture from dura showed heavy growth initially, but repeated culture on day-10 of treatment showed scant growth of the organisms. 

##### **Discussion** 

CRE infections are complicated medical condition that require multidisciplinary health care teams’ collaborative management. However, it becomes even more challenging when the site of infection involves CNS. First of all, there are not many antibiotics that providers can utilize. In addition, pharmacokinetic profiles of those drugs, especially newer ones, do not have reliable data for CNS penetration or achievable concentration for optimal antibiotic activities [9,10]. Meropenem has known distribution to cerebrospinal fluid, but the tissue distribution data for meropenem-vaborbactam or vaborbactam alone has not been well established [10]. Due to this limitation, health care providers are often forced to seek for primary literature to extrapolate the result to their patient cases. 

##### **Conclusions** 

Systemic administration of meropenem-vaborbactam showed the reduction of carbapenemase-resistant *Klebsiella pneumoniae* burden in subgaleal space. Our patient case may provide a clinical data for meropenem-vaborbactam utilization in such condition. 

###### **References** 

1. Munoz-Price, L.S.; Poirel, L.; Bonomo, R.A.; Schwaber, M.J.; Daikos, G.L.; Cormican, M.; Cornaglia, G.; Garau, J.; Gniadkowski, M.; Hayden, M.K.; et al. Clinical epidemiology of the global expansion of *Klebsiella pneumoniae* carbapenemases. *Lancet Infect Dis.*
**2013**, *13*, 785–796.
2. Iovleva, A.; Doi, Y. Carbapenem-resistant *Enterobacteriaceae. Clin. Lab. Med.*
**2017**, *37*, 303–315.
3. Doi, Y. Treatment options for carbapenem-resistant gram-negative bacterial infections. *Clin. Infect Dis.*
**2019**, *69* (Suppl. 7), S565–S575.
4. Sheu, C.C.; Chang, Y.T.; Lin, S.Y.; Chen, Y.H.; Hseuh, P.R. Infections caused by carbapenem-resistant *Enterobacteriaceae*: An update on therapeutic options. *Front. Microbiol.*
**2019**, *10*, 80.
5. Tumbarello, M.; Viale, P.; Viscoli, C.; Trecarichi, E.M.; Tumietto, F.; Marchese, A.; Spanu, T.; Ambretti, S.; Ginocchio, F.; Cristini, F.; et al. Predictors of mortality in bloodstream infections caused by *Klebsiella pneumoniae* carbapenemase-producing *K. pneumoniae*: importance of combination therapy. *Clin. Infect Dis.*
**2012**, *55*, 945–950.
6. Cho JC, Zmarlicka MT, Shaeer KM, Pardo J. Meropenem/vaborbactam, the first carbapenem/beta-lactamase inhibitor combination. *Ann. Pharmacother*. **2018**, *52*, 769–779.
7. Vabomere (meropenem and vaborbactam) [prescribing information]. Lincolnshire, IL: Melinta Therapeutics; July 2018.
8. Dai Y, Zhang C, Ma X, et al. Outbreak of carbapenemase-producing *Klebsiella pneumoniae* neurosurgical site infections associated with a contaminated shaving razor for preoperative scalp shaving. *Am. J. Infect Control.*
**2014**, *42*, 805–806.
9. Petty LA, Henig O, Patel TS, Pogue JM, Kaye KS. Overview of meropenem-vaborbactam and newer antimicrobial agents for the treatment of carbapenem-resistant *Enterobacteriaceae. Infect Drug Resist*. **2018**, *11*, 1461–1472.
10. Jorgensen, S.C.J.; Rybak, M.J. Meropenem and vaborbactam: stepping up the battle against carbapenem-resistant *Enterobacteriaceae. Pharmacother.*
**2018**, *38*, 444–461.

#### **2.8.6. Aspergillosis in an Immunocompetent Patient** 

DuarteT. IsidoroSequeiraT.GermanoN.Intensive Care Medicine Department, Curry Cabral Hospital, Central Lisbon University Hospital Center, Lisbon, Portugal

Aspergillosis is a disease caused by environmental molds Aspergillus spp. Saprophytic colonization can occur manifesting as pulmonary aspergillomas within a preexisting lung cavity, in most cases due to tuberculosis sequelae. Tissue invasion presenting as invasive pulmonary aspergillosis or disseminated infection afflicts immunocompromised and critically ill hosts.

We present a forty-three year old male, natural from Guiné-Bissau. Past history of type 1 diabetes mellitus and pulmonary tuberculosis 3 years ago treated with 6-month anti bacilar therapy. He presented abdominal pain and weight loss (~30 kg) during the last year. On examination, he had fever and a painful abdomen to palpation with no defense or signs of peritoneal irritation. Blood tests showed leukopenia, CPR 17.8 mg/dL, LDH 185 U/L, normal kidney and liver function. Chest X-ray showed an hypotransparency in the left pulmonary apex. Negative sputum samples for acidalcohol bacilli. Negative HIV and viral hepatitis serologies.

During the subsequent days he maintained the abdominal pain despite therapy with proton-pump-inhibitors and symptomatic analgesia. Upper-endoscopy showed an extensively ulcerated esophagus and stomach with mucosa edema—negative histology for *Helicobacter pylori*; positive biopsy for *Aspergillus flavus.* Normal abdomen ultrasound.

Because of gastrointestinal intolerance, he started amikacin, levofloxacin and rifampicin. Associated study with computed tomography revealed pulmonary cavitated lesions filled by soft tissue components, bilateral mycetomas and pneumatoceles; mucosal thickening of the ileus, appendix and right colon, without signs of ischemia or acute appendicitis. Bronchoscopy was performed and galactomannan antigen was positive. Bronchoalveolar culture was positive for *Aspergillus flavus*. Pulmonary invasive aspergillosis with diagnosed and amphotericin B were added. Echocardiogram excluded vegetations. Evolution to shock with multiorgan dysfunction and death on the 11th day after hospital admission.

Diabetes mellitus and active tuberculosis may cause a down-modulation in immune response, facilitating the development of fungal infections. Invasive Aspergillosis is associated with high mortality rates beside therapy with standard amphotericin B. New approaches and new therapies are needed to improve patients’ outcome.

#### **2.8.7. Combined Extracorporeal Blood Purification in Neurosurgical Patients: A Case Series** 

I.Burov А.A.Savin I.А.Abramov Т.S.Korotkov D.S.Kostritsa N.Federal State Autonomous Institution «N. N. Burdenko National Medical Research Center of Neurosurgery» of the Ministry of Health of The Russian Federation

##### **Introduction** 

Sepsis and septic shock in patients with primary brain damage of different origin can lead to or aggravate the existing cerebral and focal neurologic symptoms due to cytokine storm, impairing the hematoencephalic barrier.

The development of septic shock in the setting of low cerebral perfusion pressure aggravates the cerebral damage, which may become irreversible, leading to negative outcome or death.

In clinical situations described above the blood purification may represent a valid therapeutic option, as it aims at elimination of substances, such as cytokines, lipopolysaccharides and other Pathogen Associated Molecular Patterns from the systemic blood flow.

In neurosurgical patients with septic shock it is feasible to use a combined approach, consisting of cytokine adsorption and continuous renal replacement therapy (CRRT) with increased adsorption capacity membranes.

Of note, the combination of these methods has the potential to eliminate a wider spectrum of various substances from the blood flow in the short time.

In this study we present a case series, describing septic shock patients after neurosurgical interventions, treated with a combined blood purification therapy, including CRRT and cytokine adsorption.

##### **Objective** 

The objective of the present study is to evaluate the effectiveness of the combined extracorporeal blood purification in neurosurgical patients with septic shock.

##### **Materials and Methods** 

5 patients with septic shock were treated with the described treatment. All the patients received standard therapy according to the Surviving Sepsis Campaign guidelines. In 2 patients the combined blood purification was started within 10 h after septic shock diagnosis and antibiotic treatment initiation, the other 2 patients had the same treatment started within 20 h, and 1 patient—a day after the diagnosis and isolated CRRT initiation. The duration of the combined blood purification was 24 h.

The procedure was performed with the Prismaflex CRRT machine in the continuous venovenous hemodiafiltration mode. The systemic anticoagulation with heparin was performed in 2 patients, the other 3 patients received regional citrate anticoagulation.

In 4 patients the AN69ST increased capacity dialysis membrane was used, 1 patient had a set with lipopolysaccharide elimination capability (Oxiris). All patients had cytokines adsorber (CytoSorb) installed into the circuit after the dialysis filter.

In this case series, we traced the combined blood purification procedure influence on the hemodynamics, vasopressor requirement and organ dysfunction (by SOFA score).

##### **Results** 

In the course of the combined blood purification treatment 3 patients had substantial clinical improvements. In 24 h after combined blood purification initiation their SOFA score was reduced for 8, 3, 4; in 48 h—for 7, 2, 6; in 72 h—for 8, 2, 4 respectively.

2 patients had a transient clinical improvement: their SOFA score was reduced for 1 and 2 points in 24 h after the blood purification initiation, then for 1 in both patients at 48 h and increased by 2 points in first patient patients and decreased in 2 points in second patient in 72 h compared to start point. In both 2 patients further deterioration was according to herniation.

In 2 out of the 3 improving patients the norepinephrine demand was reduced from 0.7 and 1 to 0.00 and 0.09 µg/kg/min within 24 h from combined blood purification start; the vasopressor demand scarcely was changed in the patient in whom it was initially low (increased from 0.18 to 0.23) µg/kg/min. 

In the 2 patients with transient improvement the vasopressor requirement was reduced within 48 h from the procedure initiation (from 2.13 to 1.8 and from 0.71 to 0.15 µg/kg/min), though due to cerebral herniation the norepinephrine dose was later increased to more than 2 µg/kg/min.

The 3 improving patients were stabilized with the intensive care, including the combined blood purification. They no longer required vasopressor support, their multiorgan failure regressed. In 25 days 1 patient died of intracerebral bleeding, 2 patients were transferred to rehabilitation stable.

Patients with transient clinical improvement died in 72 and 74 h after combined blood purification start due to cerebral herniation in the setting of malignant diffuse edema.

##### **Conclusions** 

In the described case series, combined blood purification used on top of standard intensive septic shock treatment contributed to overall clinical improvement. Among the key changes were the significant vasopressor reduction and MOF regress. The use of combined blood purification in septic shock patients can be rational and effective, as it contributes to shock reversal. Further studies on the method efficacy and safety in neurosurgical patients with septic shock are required.

#### **2.8.8. The Use of Aerosolised Therapeutics in the Treatment of Ventilator Associated Infections** 

FernándezElena FernándezEainMarc Mac GiollaBennettGavinJoyceMaryMacLoughlinRonanAerogen, IDA Business Park, Dangan, Galway, Ireland.

##### **Introduction** 

Invasive ventilation is a mainstay in the treatment of severe pulmonary infections. However, a common caveat of this type of treatment is a ventilator associated respiratory infection, such as pneumonia or tracheobronchitis. The administration of aerosolised therapeutics is the most effective means of treating ventilator associated respiratory infections in mechanically ventilated patients. 

##### **Objectives** 

The objectives of this study are to assess the aerosolised characteristics of a selection of the most commonly used compounds in the treatment of ventilator associated infections and examine the effect of ventilator circuit configuration on therapeutics delivery. Two common ventilator configurations were used, one with an active humidifier and one with a heat and moisture exchanger (HME). 

##### **Methods** 

Aerosol droplet size distributions, specifically the volume mean diameter (VMD) and fine particle fraction (FPF), were measured using laser diffraction (Spraytec, Malvern, UK). The therapeutics analysed were Albuterol sulfate (Sigma Aldrich, Ireland), Promixin (Xellia Pharmaceuticals ApS, Denmark), Tobramycin (Hospira UK Ltd., UK) and Amikacin (Lab. It. Biochim. Farm.Co., Spain). Albuterol was used as a representative tracer in line with international nebuliser test standards, ISO27427. A 2 mL dose of 5 mg/mL albuterol sulfate was aerosolised using a vibrating mesh nebuliser (Aerogen Solo, Aerogen, Ireland) during simulated mechanical ventilation (Servo-U, Maquet, Sweeden) to examine the effects of ventilator circuit configuration on the delivered dose to an adult (Vt 500 mL, 15 BPM, I:E 1:1) tracheostomy patient. The first ventilator circuit arrangement had a humidifier, with the nebuliser placed on the dry side of the humidifier within the dual limb circuit (RT200, Fisher & Paykel, New Zealand), and the second circuit had a HME (Inter-Therm filtro HME, Intersurgical), attached between the patient side of the wye and the tracheostomy tube (TT) (adult ID 6.4 mm, Shiley, Medtronic, Ireland). A capture filter (Respigard, Baxter, Ireland) was placed between the TT and the artificial lung. The mass of drug delivered was determined using UV spectrophotometry at 276 nm. Results, indicating the dose delivered to the patient, are expressed as the percentage of the nominal dose placed in the nebuliser’s medication cup.

##### **Results** 

The results are shown in the tables below.

**Table 1.** Aerosol characteristics of commonly used therapeutics in the treatment of ventilator associated respiratory infections.
**Therapeutic****VMD (µm)****FPF 1–5 µm (%)****FPF < 3.5 µm (%)****Delivery Rate (mL/min)****Delivery Rate (mg/min)**Albuterol sulfate (5 g/mL)4.42 ± 0.2542.01 ± 1.240.86 ± 2.870.58 ± 0.012.91 ± 0.05Promixin (5 mg/mL)5.07 ± 0.3937.12 ± 1.338.47 ± 2.660.23 ± 0.011.61 ± 0.05Tobramycin (80 mg/mL)4.98 ± 0.1840.11 ± 1.6338.47 ± 2.320.53 ± 0.0442.64 ± 3.05Amikacin (2.5 mg/mL)3.48 ± 0.557.07 ± 2.1946.74 ± 2.770.10 ± 0.012.52 ± 0.14

**Table 2.** Effect of ventilator circuit configuration on the efficacy of aerosol delivery.
**Therapeutic****Circuit Arrangement****Lung Dose (%)**Albuterol sulfate (5 mg/mL)With Humidifier32.13 ± 1.96With HME30.96 ± 0.65

##### **Conclusions** 

These findings confirm that the majority of the aerosolised particles are within the 1–5 µm inhalable range to treat ventilator associated infections. Simulated breathing during mechanical ventilation of an adult tracheostomy patient indicates that ventilator circuit arrangement did not have a statistically significant effect on the dose delivered to the patient, with 32.13 ± 1.96% of the dose of a tracer aerosol delivered in an active humidification circuit, and 30.96 ± 0.65% delivered in a typical passive humidification (HME) circuit under the same breathing parameters (*p* = 0.206).

#### **2.8.9. An (Infectious) Year in the Life of an Ecmo Unit** 

ThomasStephanie^1^BarkerDr Julian^2^BhattacharyaRanajoy Sankar^3^1Consultant in Microbiology and clinical lead, Wythenshawe Hospital, Manchester University NHS Foundation Trust2Consultant in Critical Care Medicine, Wythenshawe Hospital, Manchester University NHS Foundation Trust3Specialty registrar, Microbiology, Wythenshawe Hospital, Manchester University NHS Foundation Trust

##### **Introduction** 

Veno-venous extracorporeal membrane oxygenation (VV-ECMO) is a technique for providing extracorporeal cardiac and pulmonary support to patients with severe, potentially reversible acute cardiac or respiratory failure, unresponsive to conventional management. The ECMO circuit bypasses and defunctions the lungs, reducing the insult caused by mechanical ventilation, whilst maintaining gas exchange. The cardiothoracic critical care unit in Wythenshawe hospital of Manchester is one of the five adult ECMO centres in the UK providing service to a wide geographical area in the north-west of England. 

##### **Objectives** 

Acute respiratory failure secondary to bacterial and viral infections is not uncommon. In previously healthy individuals these should generally be reversible. However often satisfactory response is not achieved after invasive respiratory and cardiovascular support along with appropriate antimicrobials in an intensive care setting. The role of ECMO is well established under these circumstances. However it needs expensive equipment, highly skilled team of health care professional with round the clock efficiency. Infections probably are the commonest cause of reversible respiratory failure in in ICUs however limited data is available from ECMO centres. This assimilation of year -long data from our unit in Wythenshawe hospital aims to enrich knowledge and collaboration between intensivists and infection specialists with the view to formulate strategies to prevent and treat infections while on ECMO support. Physicians already involved in ECMO care in their centres will also be able to reflect on our findings and observe how this compares with their own data.

##### **Method** 

Patient data was collected from electronic record including their date of admission, length of stay and final diagnosis. The day when the patients were initiated on ECMO support was taken as the day of admission. Some patients had more than on diagnoses. We did not endeavour into looking into the fact that which of these had been the primary cause for a particular patient being unwell. We have included the non-infection cases in the analysis as well to demonstrate the wide variety of patients being served in this centre.

##### **Results** 

Between April 2018 and April 2019 the unit has managed 27 cases on VV-ECMO majority of them presenting with respiratory failure secondary to infections. Being a regional centre we accept eligible patients for ECMO from across the trust and also transfer from neighbouring trust. Generally all the transfers are agreed in advance with the on call consultant on the unit. About half of the patients during this period came with influenza with or without complications (Figure 1) and nearly all of them stayed on ECMO support for more than a week; a third remained on support more than 3 weeks (Figure 2). Amongst the non-flu patients, all but three had diagnoses of infections in the background, mainly a variety of pneumonias. There were also two post-surgical and one patient with burn injuries who had end up being connected to ECMO support. There has been a seasonal variation in the traffic of patients and the analysis shows the service being busier during the winter months.


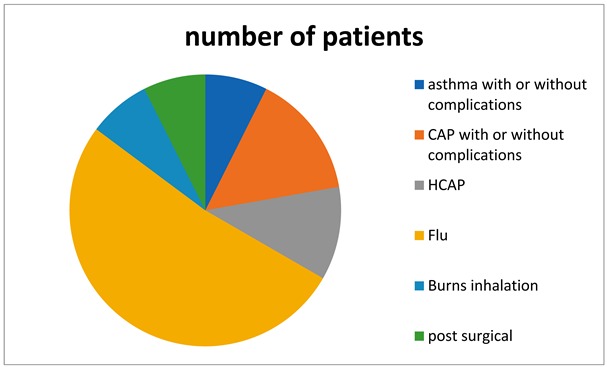


**Figure 1.** Background conditions in ECMO patients.


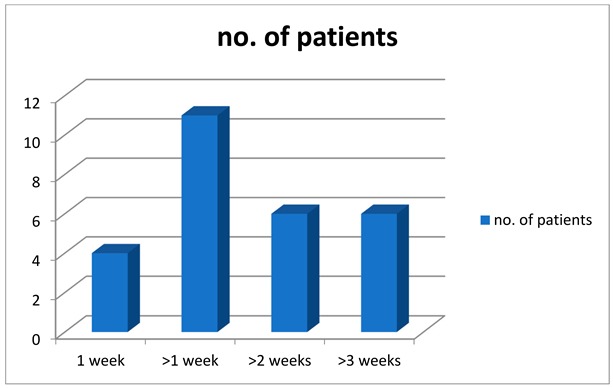


**Figure 2.** Duration over which patient stayed on ECMO.

##### **Conclusions** 

The ECMO unit in Wythenshawe hospital unit has accepted patients for ECMO support throughout the year but the traffic was disproportionately busy during the winter months. Influenza makes otherwise healthy people very unwell due to severe respiratory failure reflected on the number of flu patients managed on ECMO over the period of interest. 

#### **2.8.10. The Introduction of an Intensive Care Sepsis Checklist** 

WickramasingheManojSt James’ University Hospital, James Beck

##### **Introduction** 

Sepsis is a ubiquitous condition within critical care that is associated with high morbidity and mortality^1^. There is an abundance of literature suggesting early identification and management of sepsis is crucia^2—^on the back of this the Surviving Sepsis Campaign have made several recommendations^3^. 

Patients with sepsis are often complex with other concomitant pathologies. Therefore, it can be easy for clinicians to overlook key investigations and management steps for sepsis. There is a wealth of evidence that suggests the use of checklists within ICU can improve patient care and implementation of best evidence-based practice^4,5^. Key sepsis bundles/checklists that have been created include the Sepsis Six which was later named the BUFALO checklist^6^. 

Within our Intensive Care at St James’ University Hospital (SJUH) in England, we have recognised that there is a need for a standardised approach to the management of our septic patients. Although the BUFALO checklist, which is used on the wards, has key components in the management of sepsis, it does not capture some specifics within ICU—these include the ideal use of vasopressors, cardiac monitoring, specific investigations such as procalcitonin and the use of steroids for septic shock. 

Anecdotally we have noticed that some significant components of sepsis management can be missed septic patients. We propose to formally evaluate our practice within SJUH and then introduce an ICU-specific Sepsis Checklist.

##### **Objectives** 

Evaluate current practice within SJUH

Introduce ICU-specific Sepsis Checklist 

Improve investigation and management of patients with sepsis 

##### **Method** 

Prospective data collection for a 4-week period prior to implementation of checklist. Patients were recognised to have sepsis using the qSOFA criteria^7^. Data was anonymised and entered into an excel sheet. The checklist was introduced after the initial 4-week period and highlighted in handover and also via email to nursing and medical teams. Individual bedside explanation and highlighting of the checklist was carried out over a 1-week period to nurses. Following this data was collected during a 6-week period. 

##### **Results** 

- 30 patients identified prior to introduction of checklist
- 36 patients after introduction of checklist
- 70% adherence to use of checklist with patients with sepsis
- Prior to introduction of checklist, use of vasopressors, fluid and antibiotic prescription was good
- Key differences after checklist was implemented included blood cultures, sputum cultures, atypical pneumonia screen, baseline procalcitonin and HIV (See Table 1 and Figure 1)

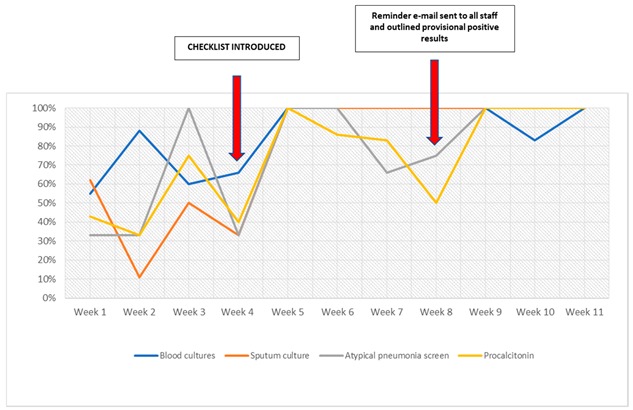


**Figure 1.** Key data before and after checklist implementation.

**Table 1.** Data with and without checklist.
**Investigation****Without Checklist****With Checklist**Blood culture66%100%Sputum culture 44%100%Atypical pneumonia 57%100%Procalcitonin43%97%HIV30%73%Urine culture 66%100%Steroids50%100%Cardiac monitoring60%100%

##### **Conclusions** 

An ICU-specific Sepsis Checklist has been well received in a tertiary unit in England. We have observed significant and sustained improvement in sepsis management since the introduction of the checklist. This reflects a large body of evidence that suggests checklists in ICU improve patient care and management. There is scope for this checklist to be rolled out across multiple critical care units.

###### **References** 

1. Martin, G.S.; Mannino, D.M.; Eaton, S.; Moss, M. The epidemiology of sepsis in the United States from 1979 through 200. *N. Engl. J. Med.*
**2003**, *348*, 1546–1554.
2. Damiani, E.; Donati, A.; Serafini, G.; Rinaldi, L.; Adrario, E.; Pelaia, P.; Busani, S.; Girardis, M. Effect of performance improvement programs on compliance with sepsis bundles and mortality: A systematic review and meta-analysis of observational studies. *PLoS ONE*
**2015**, *10*, e0125827.
3. Rhodes, A.; Evans, L.E.; Alhazzani, W.; Levy, M.M.; Antonelli, M.; Ferrer, R.; Kumar, A.; Sevransky, J.E.; Sprung, C.L.; Nunnally, M.E.; et al. Surviving sepsis campaign: International guidelines for management of sepsis and septic shock: 2016. *Crit. Care Med.*
**2017**, *45*, 486552.
4. Pronovost, P.; Needham, D.; Berenholtz, S.; Sinopoli, D.; Chu, H.; Cosgrove, S.; Sexton, B.; Hyzy, R.; Welsh, R.; Roth, G. An intervention to decrease catheter-related bloodstream infections in the ICU. *N. Engl. J. Med*. **2006**, *355*, 2725–2732.
5. Leisman, D.E.; Doerfler, M.E.; Ward, M.F.; Masick, K.D.; Wie, B.J.; Gribben, J.L.; Hamilton, E.; Klein, Z.; Bianculli, A.R.; Akerman, M.B.; et al. Survival benefit and cost savings from compliance with a simplified 3-h sepsis bundle in a series of prospective, multisite, observational cohorts. *Crit. Care Med.*
**2017**, *45*, 395406.
6. Daniels, R.; Nutbeam, T.; McNamara, G.; Galvin, C. The sepsis six and the severe sepsis resuscitation bundle: A prospective observational cohort study. *Emerg. Med. J*. **2011**, *28*, 459–460.
7. Marik, P.E.; Taeb, A.M. SIRS, qSOFA and new sepsis definition. *J. Thorac Dis*. **2017**, *9*, 943–945, doi:10.21037/jtd.2017.03.125.

